# Tumor Exosomes Reprogrammed by Low pH Are Efficient Targeting Vehicles for Smart Drug Delivery and Personalized Therapy against their Homologous Tumor

**DOI:** 10.1002/advs.202002787

**Published:** 2021-03-16

**Authors:** Changguo Gong, Xiao Zhang, Min Shi, Feng Li, Shuang Wang, Yan Wang, Yugang Wang, Wei Wei, Guanghui Ma

**Affiliations:** ^1^ Department of Gastroenterology Tongren Hospital Shanghai Jiao Tong University School of Medicine No. 1111 Xianxia Road, Changning District Shanghai 200336 P. R. China; ^2^ State Key Laboratory of Biochemical Engineering Institute of Process Engineering Chinese Academy of Sciences No. 1 Bei‐Er‐Tiao, Zhong‐Guan‐Cun, Haidian District Beijing 100190 P. R. China; ^3^ School of Chemical Engineering University of Chinese Academy of Sciences No. 19A Yuquan Road Beijing 100049 P. R. China

**Keywords:** combination therapy, lipids rearrangement, low pH reprogramming, patient‐derived xenografts, tumor exosomes

## Abstract

As membrane‐bound extracellular vesicles, exosomes have targeting ability for specific cell types, and the cellular environment strongly impacts their content and uptake efficiency. Inspired by these natural properties, the impacts of various cellular stress conditions on the uptake efficiency of tumor iterated exosomes are evaluated, and low‐pH treatment caused increased uptake efficiency and retained cell‐type specificity is found. Lipidomics analyses and molecular dynamics simulations reveal a glycerolipid self‐aggregation‐based mechanism for the enhanced homologous uptake. Furthermore, these low‐pH reprogrammed exosomes are developed into a smart drug delivery platform, which is capable of specifically targeting tumor cells and selectively releasing diverse chemodrugs in response to the exosome rupture by the near‐infrared irradiance‐triggered burst of reactive oxygen species. This platform exerts safe and enhanced antitumor effects demonstrated by multiple model mice experiments. These results open a new avenue to reprogram exosomes for smart drug delivery and potentially personalized therapy against their homologous tumor.

## Graphical abstract

Tumor exosomes treated by low‐pH condition can enhance their uptake efficiency and retain cell‐type specificity for their parent cells based on a glycerolipid self‐aggregation‐based mechanism. They are developed as a smart drug delivery platform, specifically targeting tumor cells and selectively releasing diverse chemodrugs in response to the near‐infrared irradiance, to achieve safe and enhanced antitumor effects.

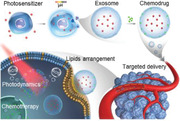

## 1 Introduction

Exosomes are membrane‐bound extracellular vesicles released by most eukaryotic cells, with a size ranging from 40 to 100 nm.^[^ ^1^ ^]^ They have been found with the capacity of transferring not only membrane components but also nucleic acid between cells, emphasizing their role in intercellular communication.^[^ ^2^, ^3^ ^]^ Furthermore, a number of in vitro and in vivo studies have also highlighted their unique targeting capabilities.^[^ ^4^, ^5^, ^6^ ^]^ That is, exosomes are able to overcome natural barriers and utilize endogenous mechanisms for uptake, intracellular trafficking, and subsequent delivery of their content in parent cells.^[^ ^7^, ^8^, ^9^, ^10^ ^]^ Accordingly, there is considerable excitement for the biotechnological applications of utilizing exosomes as drug carriers for the target delivery of various therapeutic agents to highly specific cells.^[^ ^11^, ^12^ ^]^

Recently, studies have indicated that the local cellular environment where exosomes are produced can strongly impact their cell–cell communication performance.^[^ ^13^, ^14^, ^15^, ^16^ ^]^ For example, tumor hypoxia microenvironment could increase the release of exosomes, which contributed to tumor angiogenesis and metastasis.^[^ ^17^, ^18^, ^19^ ^]^ Acidic microenvironment could also impact the properties of exosomes, which played a key factor for tumor progression.^[^ ^20^, ^21^, ^22^ ^]^ In light of this, we speculated it plausible that manipulation of culture conditions should allow the performance tuning of exosomes for delivery applications, and the screening for a superior condition, which, to the best of our knowledge, had yet been investigated. In addition to the target delivery, another wave of efforts for drug carrier has been spurred on the design in response to specific external triggers (e.g., light, sound, heat, and magnetic fields), enabling the selective release of drug at the lesion site.^[^ ^23^, ^24^, ^25^, ^26^, ^27^, ^28^, ^29^, ^30^, ^31^ ^]^ In this aspect, we further speculated it was valuable to develop a smart delivery platform with exosomes, which could further increase their targeting efficiency and reduce side effects.

**Figure 1:**
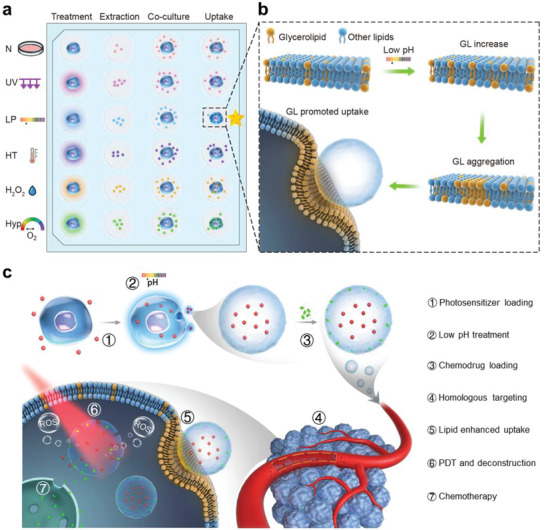
Culture conditions and mechanisms for enhancing exosome's cell uptake and its antitumor therapy applications. a) Procedure for screening cell culture conditions to obtain exosomes with high delivery efficiency, including normal condition (N), ultraviolet irradiation stress treatment (UV), low‐pH culture medium treatment (LP), high temperature treatment (HT), H_2_O_2_ treatment (H_2_O_2_), and hypoxia environment treatment (Hyp). b) Mechanism for the enhanced uptake of the lipid‐reprogrammed exosomes. c) Strategy for drug loading and synthetically anticancer therapy combining chemotherapy with photodynamic therapy.

Aiming to the anticancer drug delivery, we herein initially screened the impacts of six different cellular stress conditions on the uptake efficiency of exosomes released by the human gastric cancer cell line (**Figure** **1**a). We found that shocking cells with approximate tumor microenvironment conditions (e.g., low pH and hypoxia) caused a significant increase in tumor exosome uptake into parent cells, and the most prominent enhancement under low‐pH treatment did not reduce the cell‐type specificity at the animal level. Upon further discovery of altered lipid composition during the low‐pH reprogramming, we explored this notion in detail using molecular dynamics simulations and revealed that glycerolipids′ self‐aggregation between tumor cells and their iterated exosomes accounted for the enhanced homologous uptake (Figure 1b). Based on these findings and low‐pH reprogrammed exosomes, we further developed a smart drug delivery platform by co‐loading the hydrophilic photosensitizer and hydrophobic chemodrug in the lumen and membrane of exosomes, respectively (Figure 1c). After intravenous injection, these exosomes exerted their targeting capacity to tumor site. With further near‐infrared (NIR) laser irradiation, photosensitizer could generate reactive oxygen species (ROS) for exosome rupture and subsequent chemodrug release, leading to enhanced antitumor effects with few abnormalities (Figure 1c). Using patient‐derived xenografts (PDX) tumor model, we finally confirmed that exosomes released by primary cells from patient tumor could also promote targeted delivery efficiency to tumor area after low‐pH reprogramming, and achieve promising therapeutic effects in terms of the smart and personalized drug delivery platform.

## 2 Results

### 2.1 Screening with Various Stresses Highlights Benefits of Low‐pH Shock Treatment

**Figure 2:**
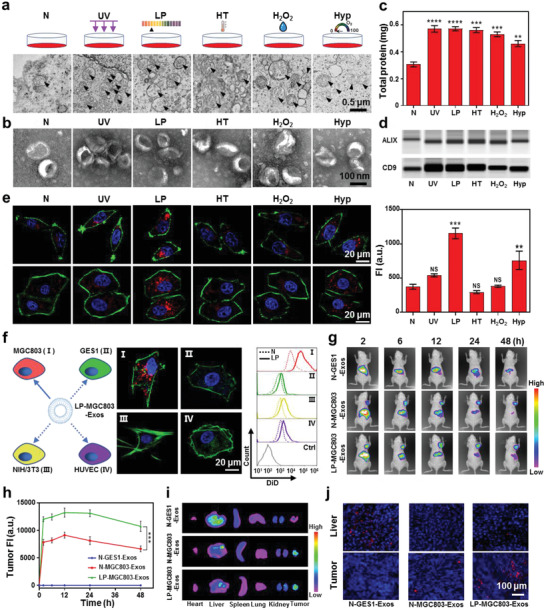
Screening diverse culture treatments in search of increased uptake efficiency for released exosomes and evaluation of their targeting specificity. a) TEM images of the differentially treated MGC803 cells. Black arrows: multivesicular bodies. b) TEM images of exosomes released by the differentially treated MGC803 cells. c) BCA protein concentration detection of the differentially treated exosome types. d) Immunoblotting with an antibody against ALIX (upper panel), and against CD9 (lower panel) of the differentially treated exosome types. e) CLSM images of differentially treated MGC803 exosome types uptake by MGC803 cells (upper panel) and HepG2 exosome types uptake by HepG2 cells (lower panel), and fluorescence intensity (FI) (from DiD labeled exosomes) in MGC803 cells. f) CLSM images of MGC803 exosomes treated by low pH (LP‐MGC803‐Exos) uptake by different types of cells (MGC803, GES1, NIH/3T3, and HUVEC) (left panel), and the intracellular fluorescence intensity (from DiD labeled exosomes) determined using flow cytometry, compared with the N‐MGC803‐Exos (right panel). g) In vivo distributions of N‐GES1‐Exos, N‐MGC803‐Exos, and LP‐MGC803‐Exos in MGC803 tumor bearing BALB/c nude mice at different time points post intravenous injection. h) Quantitative time‐dependent distributions of N‐GES1‐Exos, N‐MGC803‐Exos, and LP‐MGC803‐Exos at MGC803 tumor sites. i) Ex vivo fluorescence images of main organs. j) Frozen sections of livers and tumors; nuclei and exosomes are indicted with blue and red fluorescence, respectively. Data in (c), (e), and (h) represent mean values ± SD, *n* = 3. Statistical differences were determined by one‐way ANOVA test. NS means no significant difference. **p < 0.01, ***p < 0.001, ****p < 0.0001.

To screen the opportunities of alerting culture conditions for exosome's higher uptake efficiency, we cultured MGC803 human gastric cancer cells with diverse treatments, including normal conditions (N), ultraviolet irradiation stress treatment (UV; 40 W), low‐pH culture medium treatment (LP; pH 4.0), high temperature treatment (HT; 40 °C), H_2_O_2_ treatment (H_2_O_2_; 250 × 10^−6^ m), and hypoxia environment treatment (Hyp; 100% N_2_). After these treatments, the cells were confirmed with good viability and further examined with transmission electron microscopy (TEM). Compared with the N‐group, all of the treated cells had significantly increased numbers of multivesicular bodies (**Figure** **2**a), which are the typical sites of exosome genesis.^[^ ^32^ ^]^

We then used differential centrifugation to separate the exosomes sourced from these treated cells, as described in the Experimental Section. TEM images revealed that exosomes from all of the treated cells maintained apparently normal morphology (Figure 2b), and nanoparticle tracking analysis (NTA) showed size distributions for all exosomes centered around 100 nm with three replicates (Figure S1, Supporting Information). We examined the total protein content of exosomes released from the same amount (1.5 × 10^7^) of cells via the bicinchoninic acid (BCA) assay kit to represent their accurate quantitation for each of the treatments. Compared with the normal exosomes (N‐Exos), there was significantly increased total exosome protein content in the samples of the various treatments (Figure 2c). More specifically, these treated samples were also detected with increased accumulation of the exosome marker proteins ALIX and CD9 (Figure 2d).

In addition to the improved production, we further paid attention to exosomes uptake by their parent cells. To this end, MGC803 cells were treated with different exosomes (labeled with fluorescent dye DiD), and the uptake behaviors were analyzed by the confocal laser scanning microscopy (CLSM) and flow cytometry. As shown in Figure 2e, we observed significant differences in the uptake efficiency among the exosomes released by variously treated cells. The low‐pH and hypoxia stimulated exosomes (i.e., LP‐Exos and Hyp‐Exos, respectively) exhibited the higher uptake efficiency (Figure 2e), which indicated that the approximate tumor microenvironment conditions could improve exosome's uptake properties. Above trends were also observed on HepG2 human liver cancer cells, indicating the wide applicability of this approach to reprogram exosomes with superior production and targeting capacity (Figure 2e and Figure S2, Supporting Information). We then focused on the most prominent LP treatment, and found such an enhanced uptake was gradually compromised with the pH value of culture medium increasing from 4.0 to 7.4 (Figure S3, Supporting Information), which guided us to use pH 4.0 treatment to produce LP‐Exos for subsequent experiments.

### 2.2 Low‐pH Reprogramming of Exosomes Substantially Enhances Their Tumor Targeting Specificity

After demonstrating the improved uptake of LP‐Exos by their parent cells, we further evaluated whether this benefit was specific. To this end, LP‐Exos (labeled with DiD) released from MGC803 cells were exposed to diverse cells, and their uptake characteristics were evaluated by the CLSM. As shown in Figure 2f, LP‐MGC803‐Exos were specifically endocytosed by MGC803 cells, whereas the other cell types (GES1 human gastric mucosa epithelial cells, NIH/3T3 mouse embryo fibroblast cells, and HUVEC human umbilical vein endothelial cells) had very little uptakes. For further verification, the uptakes of both N‐MGC803‐Exos and LP‐MGC803‐Exos to above diverse cells were also comparatively investigated by flow cytometry (Figure 2f). Although superior uptake of LP‐MGC803‐Exos over that of N‐MGC803‐Exos was observed for each cell type, we did notice a much more improvement for MGC803 cells, again indicating a specific targeting behavior to their parent cells.

To determine whether such MGC803 exosome targeting specificity also occurred in vivo, we inoculated MGC803 cells into the oxter of BALB/c null nude mice to form a subcutaneous tumor xenograft, and injected diverse fluorescence‐labeled exosomes into mice through the tail vein. We monitored the fluorescence signal distribution at different time points of a 48 h post‐injection time course by CLSM for N‐GES1‐Exos, N‐MGC803‐Exos, and LP‐MGC803‐Exos, respectively. In vivo imaging revealed that N‐GES1‐Exos were persistently accumulated at the liver area, with no transfer to tumors. Owing to the homology, the signal of N‐MGC803‐Exos at tumor area could gradually increase within 48 h, while gradually decrease at liver. With the low‐pH reprogramming, LP‐MGC803‐Exos exhibited similar trends with the N‐MGC803‐Exos, but had a faster migration rate and a stronger intensity at tumor area (Figure 2g,h). Ex vivo fluorescence imaging and frozen sections of tumors and organs (heart, liver, spleen, lung, and kidney) were also consistent with the in vivo imaging results (Figure 2i,j and Figure S4, Supporting Information), which further supported that the exosomes had benefited from the low‐pH reprogramming for the enhanced homologous tumor targeting in vivo.

### 2.3 The Glycerolipids′ Self‐Aggregation Accounts for the Enhanced Homologous Targeting

**Figure 3:**
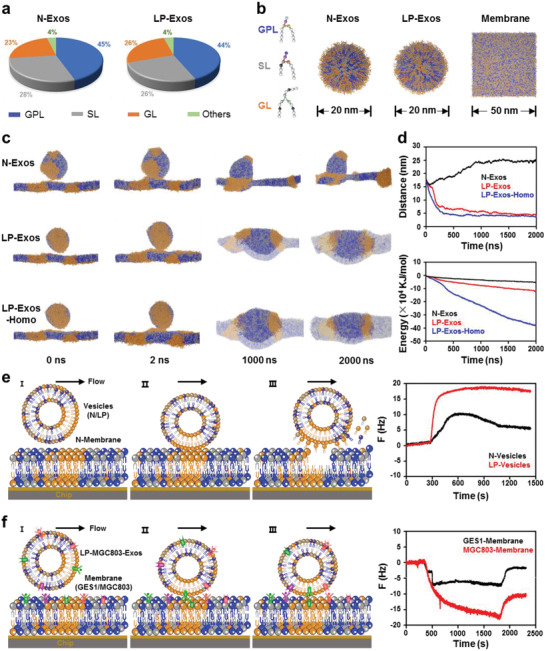
Molecular mechanism for enhanced uptake of LP‐Exos. a) Lipidomics data for N‐Exos and LP‐Exos. b) The corresponding initial models for these two types of exosomes and cell membrane in molecular dynamics simulations. c) Typical structure diagrams for 2 µs interaction simulations of N‐Exos, LP‐Exos, and the homologous targeting LP‐Exos‐Homo with cell membrane, respectively. d) Characteristic centroid distance and energy between exosome and membrane for N‐Exos, LP‐Exos, and LP‐Exos‐Homo during the simulation process. e) Schematic illustration for QCM experiments using artificial vesicles (N‐Vesicles or LP‐Vesicles) constituted by three lipids in accordance with the simulations to flow across a lipid membrane (spin‐coated with N‐Vesicles) on the chip (left panel) to verify the advantage of LP treatment, and corresponding QCM curves (right panel). I: Start pumping vesicles; II: Vesicles contact and interact with the lipid membrane; III: Vesicles detach from the lipid membrane, accompanied by extracting membrane lipids which were slightly adhered to the chip. f) Schematic illustration for QCM experiments using LP‐MGC803‐Exos to flow across a cell membrane (spin‐coated with GES1 or MGC803 cell membrane) on the chip (left panel) to verify the homologous targeting, and corresponding QCM curves (right panel). I: Start pumping exosomes; II: Exosomes contact and interact with the cell membrane; III: Exosomes are stably adsorbed on the cell membrane which was tightly adhered to the chip.

Based on the observed enhanced homologous uptake of LP‐Exos, we sought to elucidate the underlying mechanism. Considering the facts that external environment could strongly impact exosome components,^[^ ^33^ ^]^ and exosome membrane could directly mediate its cellular uptake,^[^ ^34^ ^]^ we thus focused on the membrane components. We first conducted proteomics analyses and found no significant differences of uptake‐associated membrane proteins (such as actin‐related proteins, cadherin proteins) between N‐Exos and LP‐Exos, which excluded the role of membrane protein on the enhanced homologous uptake of LP‐Exos. In this case, we further performed lipidomics analyses, and the data showed the lipid composition of both N‐Exos and LP‐Exos was mainly glycerophospholipid (GPL), sphingolipid (SL), glycerolipid (GL), which together dominated 96% of total lipids. The others 4% lipids included steroids and derivatives, prenol lipids, and fatty acyls. Compared with N‐Exos, the LP‐Exos altered proportions of GPL, SL, and GL, with about 1% less GPL, 2% less SL, and 3% more GL (**Figure** **3**a). More detailed comparisons on the subtypes of GPL, SL, and GL also showed the corresponding alterations (Figure S5, Supporting Information). We speculated that such distinct lipids composition may account for the observed differences in the uptake properties of the two types of exosomes.

To confirm this speculation, we performed a series of molecular dynamics simulations to explore the molecular interactions occurring between exosomes and cells. We simplified exosomes to the initial models of N‐Exos and LP‐Exos, which were consisted of GPL, SL, and GL dominant subtypes in the proportions from our lipidomics data (Figure 3b). Cells were simplified to a lipid membrane, whose composition was consistent with N‐Exos (Figure 3b). These lipids in models were initially evenly distributed and then 1 µs equilibrium simulations were conducted to stabilize the structures of these models. The terminal structures of equilibrium simulations were extracted and subsequently integrated for a 2 µs interaction simulations between each type of exosomes and membrane, using the Martini force field.^[^ ^35^, ^36^ ^]^ The interaction simulations could mimic the exosome–cell interactions, and revealed the underlying mechanism.

Typical structure diagrams for the simulation process of N‐Exos and LP‐Exos indicated that GL lipids underwent self‐aggregation during the simulated equilibrium process (0 ns), which could be attributed to their stronger hydrophobicity compared with GPL and SL lipids. Induced by this situation, both exosome types rapidly connected with membranes within 2 ns. After this initial connection, GL lipids in both the exosome and the membrane experienced local aggregation, concentrated at the interface between the exosome and the membrane until 2000 ns, with the system having reached a relatively stable state (Figure 3c). However, there were apparent distinctions between N‐Exos and LP‐Exos: N‐Exos could not be completely enveloped by the membrane during 2000 ns simulation, while the LP‐Exos achieved complete membrane encapsulation (Figure 3c). Such a distinct dynamic behavior implied that the increased GL lipids of LP‐Exos promoted the cellular uptake for exosomes. The corresponding distance and energy curves between the two exosome types and the membrane validated the functional contribution of the increased GL lipids, with LP‐Exos having both complete endocytosis and higher interactional energy (Figure 3d).

Based on the above simulations, we further explored the underlying mechanism for homologous targeting of exosomes. Considering that this specificity is mainly due to the interactions of receptors and ligands on the surface of exosomes and cells, we thus modified the parameters of beads that constituted the GL lipids for strengthening the interaction between exosomes and membrane to mimic the homologous targeting capacity of exosomes. As shown in Figure 3c, typical structure diagrams for the simulation process of LP‐Exos‐Homo shared a similar trend as the LP‐Exos, which also eventually achieved complete membrane encapsulation. Notably, the corresponding distance and energy curves displayed that LP‐Exos‐Homo had a shorter overall endocytosis durations time and a higher interactional energy (Figure 3d), which supported an endocytosis‐promoting contribution from homologous targeting.

Above simulations provided a plausible GL‐lipid self‐aggregation and targeting specificity‐based hypothetical explanation for the observed differences in the distinct endocytosis efficiency of MGC803 exosomes. To further validate these simulation results, we first conducted a series of quartz crystal microbalance (QCM) experiments to reproduce the simulated interfacial interactions. Two types of vesicles constituted by three lipid components (GPL, SL, and GL dominant subtypes) in the proportions of above simulations were prepared with the size of about 200 nm, and the mixed lipid components of N‐Vesicles were paved on the chip to mimic the cell membrane. QCM curves showed that the interaction strength of GL‐lipid self‐aggregation between vesicles and membrane was stronger than that of lipids′ physical absorption among membrane, and the relatively GL‐lipid‐rich LP‐Vesicles indeed exhibited a higher binding efficiency with membrane, which resulted in more detachment of lipids from the layer on the chip (Figure 3e). To verify the homologous targeting, a more physiological QCM experiment was performed in which GES1 or MGC803 cell membrane was extracted and paved on the chip, and LP‐MGC803‐Exos were prepared for interaction. The frequency curves showed that LP‐MGC803‐Exos displayed a higher adhesion efficiency with MGC803 cell membrane (nearly three times of GES1 cell membrane) (Figure 3f). Moreover, the strong interfacial interaction captured the LP‐MGC803‐Exos on the cell membrane, which resulted in increased mass (Figure 3f). These data suggested that the high content of GL‐lipid and the homologous targeting indeed produced stronger interface interactions.

Encouraged by above results, we further verified the contributions of GL on exosome's uptake efficacy. We constructed three kind of vesicles with a concentration gradient of GL (25%, 50%, 75%) for magnifying GL's effects and co‐cultured them (labeled with DiD dye) with MGC803 cells. CLSM and flow cytometry analyses showed that the vesicles with 75% GL exhibited the highest uptake efficiency, and the vesicles with 25% GL exhibited the lowest uptake efficiency (Figure S6, Supporting Information), which all demonstrated that the ratio of GL was indeed very important for exosome's uptake. Collectively, these simulations and confirmatory experimental results illustrated how the differential lipid composition and homologous interaction of LP‐Exos contributed to their enhanced cellular uptake by parent cells after low pH reprogramming. Meanwhile, considering the fact that exosomes lack nucleus and organelles which are essential for lipid bilayer synthesis, the resulting LP‐Exos will remain their low‐pH reprogrammed lipid composition upon any pH microenvironment, which can be further well‐designed as smart drug delivery system for cancer therapy.

### 2.4 Construction of an LP‐Exos‐Based Smart Drug Release Platform for Combination Therapy

**Figure 4:**
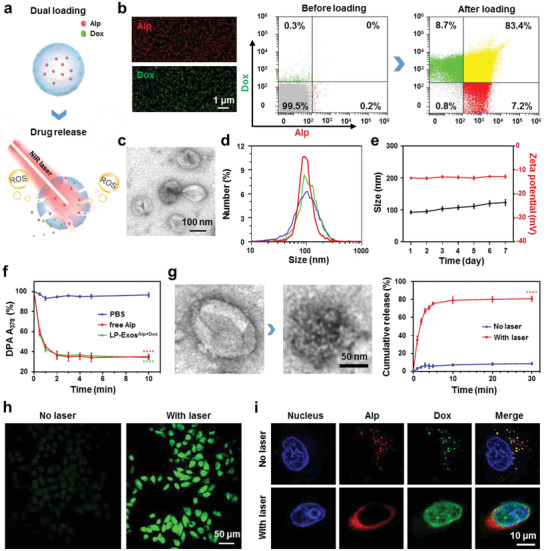
Characterizations of drug‐loaded LP‐Exos and evaluation of laser‐controlled drug release. a) Schematic illustration of dual‐loaded LP‐Exos (upper panel) and laser‐triggered drug release from LP‐Exos (lower panel). Red: Alp; Green: Dox. b) CLSM and flow cytometry analyses of the dual‐loaded LP‐Exos. c) TEM images of the dual‐loaded LP‐Exos. d) Size distributions of the dual‐loaded LP‐Exos with three replicates. e) A typical variation in the average sizes (black line) and zeta potentials (red line) of the dual‐loaded LP‐Exos in PBS over 7 days. f) Evolution of decay curves for the relative absorption of DPA at 378 nm for the different treatments. Blue: PBS; Red: PBS+free Alp; Green: PBS+LP‐Exos^Alp+Dox^. g) TEM images of normal and ruptured exosomes (left panel), and spectrophotometry measuring absorption at 480 nm wavelength showing cumulative release percentage of Dox from LP‐Exos^Alp+Dox^ with or without laser irradiation (right panel). h) Generation of ROS in the cytoplasm of MGC803 cells co‐cultured with LP‐Exos^Alp+Dox^ using DCFH‐DA probe before and after laser irradiation. i) CLSM images for the localizations of Dox (green) and Alp (red) after LP‐Exos^Alp+Dox^ uptake by MGC803 cells, with or without laser irradiation. Data in (e), (f), (g) represent mean values ± SD, *n* = 3. Statistical differences in (f) were determined by one‐way ANOVA test. Statistical differences in (g) were determined by two‐tailed student's *t*‐test. ^****^ *p* < 0.0001.

Based on the improved uptake and targeting specificity of LP‐Exos, we continued to develop the LP‐Exos for smart anticancer therapy by exploring a dual‐loading strategy. Briefly, a hydrophilic photosensitizer compound Al(III) phthalocyanine chloride tetrasulfonic acid (Alp) was loaded into the LP‐Exos lumen by co‐cultured with tumor cells in low‐pH culture medium, and then LP‐Exos^Alp^ released by cells were loaded the hydrophobic chemodrug doxorubicin (Dox) into the lipid membrane by 1 h incubation at 37 °C, obtaining LP‐Exos^Alp+Dox^ (**Figure** **4**a). Upon the enhanced tumor targeting, LP‐Exos^Alp+Dox^ were speculated to efficiently generate singlet oxygen by NIR irradiation of Alp, which could not only deconstruct the exosome for selectively releasing Dox to induce chemotherapy, but also enable the photodynamics for combination therapy (Figure 4a).

After the preparation of LP‐Exos^Alp+Dox^, we first examined the loading conditions of these two molecules. CLSM and flow cytometry analyses showed that Alp and Dox were successfully dual‐loaded in the LP‐Exos (Figure 4b), with the calculated Alp's loading rate being ≈33% and the Dox's loading rate being ≈13% (Figure S7a, Supporting Information). Using TEM imaging and NTA, we then characterized the LP‐Exos^Alp+Dox^ and found that compared to the blank LP‐Exos, the dual‐loading had almost no effects on their morphology, size, and potential distributions (Figure 4c,d and Figure S7b, Supporting Information). Supporting the attractive stability of the LP‐Exos^Alp+Dox^, NTA analyses showed no significant changes in the particle sizes and zeta potentials for freshly prepared LP‐Exos^Alp+Dox^ under different treatments, which were stored in phosphate buffered saline (PBS) for 1 week, or rehydrated after lyophilization, respectively (Figure 4e and Figures S7c and S8, Supporting Information). Note that the above construction and detection analyses of the exosome‐based smart drug carrier LP‐Exos^Alp+Dox^ were repeated for three times, which proved the superior reproducibility of the engineering process.

To assess the laser irradiation effects for generating singlet oxygen, we also performed a standard spectrophotometry‐based assay, using a reporter reagent 9,10‐diphenylanthracene (DPA).^[^ ^37^ ^]^ Upon irradiation with a 660 nm wavelength laser at 1 W power, the LP‐Exos^Alp+Dox^ could significantly produce singlet oxygen to decrease the DPA signal within 2 min, and the presence of LP‐Exos did not affect the nature of Alp (Figure 4f). Such an efficient ROS production indeed changed the morphology of LP‐Exos (Figure 4g), indicating the occurrence of rupture. As a result, the released Dox rapidly increased upon the laser excitation, eventually reaching a stable level within 5 min (Figure 4g), which demonstrated an effective responsive release.

For verification of above ROS production, LP‐Exos rupture, and responsive release, we further evaluated these properties of LP‐Exos^Alp+Dox^ in MGC803 cells. On the one hand, we used the 2′,7′‐dichlorodihydrofluorescein diacetate (DCFH‐DA), a commercially available dye typically underwent ROS‐mediated conversion from nonfluorescent to fluorescent form, to monitor NIR‐laser‐triggered singlet oxygen production in MGC803 cells after co‐culture for 30 min and laser excitation for 3 min. CLSM imaging showed significantly increased intensity upon laser irradiation (Figure 4h), which suggested that Alp had been triggered to transform molecular oxygen into plentiful singlet oxygen. On the other hand, we also used CLSM to examine how the laser‐irradiation‐triggered rupture of LP‐Exos^Alp+Dox^ inside MGC803 cells affected the localization of Dox. Prior to irradiation, Alp and Dox were co‐localized outside of the nucleus. Upon laser‐irradiation‐triggered rupture, the co‐localization disappeared: Dox entered the nucleus for targeting the DNA, and Alp was around the nucleus for oxidation damage (Figure 4i). These results together demonstrated that LP‐Exos^Alp+Dox^ could behave in MGC803 cells as the predetermined design idea.

### 2.5 LP‐Exos^Alp+Dox^ Exhibit Enhanced Tumor Cell Killing Effects In Vitro and In Vivo

**Figure 5:**
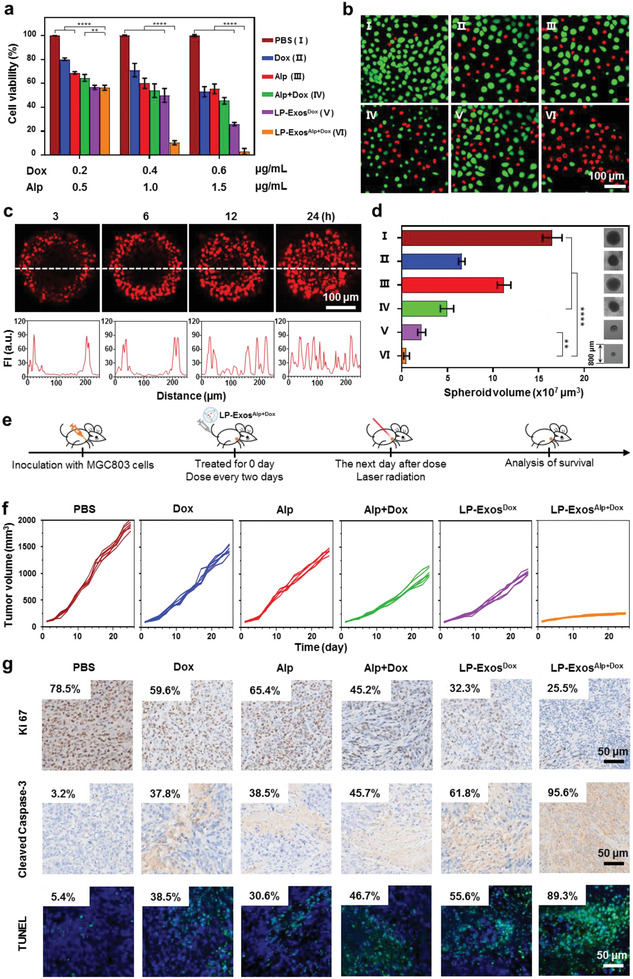
Anticancer therapy effects of the dual‐loaded LP‐Exos strategy. a) CCK‐8 cytotoxicity analysis of MGC803 cells given six treatment types, including PBS, Dox, Alp irradiated by laser (Alp), Alp and Dox irradiated by laser (Alp+Dox), Dox in LP‐Exos (LP‐Exos^Dox^), and Alp and Dox in LP‐Exos irradiated by laser (LP‐Exos^Alp+Dox^). Three different dose strengths were tested for each treatment. The doses of Dox and Alp were calculated from the loading rate in the approximate proportion of 2:5. b) Live/dead analysis after 24 h culture for MGC803 cells under the six treatments. Green: live cells; Red: dead cells. c) CLSM images (upper panel) and line fluorescence intensity analysis (lower panel) for LP‐Exos^Alp+Dox^ penetration in MGC803 cell spheroids at different time points. d) Volumes of the MGC803 cell spheroids with the six treatments after co‐incubation of 96 h and the typical images for MGC803 cell spheroids. e) Schematic diagram for constructing MGC803‐derived tumor xenografts and the experimental design for the six treatment types (injected into mice via tail vein, and irradiated by 660 nm and 1 W laser for 3 min). f) Evolutions of tumor volumes for MGC803‐derived tumor xenograft treated by six diverse treatments, each group contained six mice. g) Immunohistochemical analysis and quantification of cell proliferation by Ki 67 (upper panel) and cell apoptosis by Cleaved Caspase‐3 (middle panel) and TUNEL (lower panel) in MGC803 tumor tissue at the end of treatment. Positive cells calculated as the percentage of total cells. Data in (a) (*n* = 3) and (d) (*n* = 5) represent mean values ± SD. Statistical differences were determined by one‐way ANOVA test. **p < 0.01, ^****^ *p* < 0.0001.

To evaluate the anticancer effects of LP‐Exos^Alp+Dox^, we first cultured MGC803 cells with Dox and/or Alp at a concentration ratio of 2:5 according to their loading rates, with the tested Dox concentrations being 0.2, 0.4, and 0.6 µg mL^−1^ and the Alp concentrations being 0.5, 1.0, and 1.5 µg mL^−1^. Six different treatment types at each concentration of Dox and Alp were investigated, including PBS, Dox alone, Alp irradiated by laser (Alp), Alp and Dox irradiated by laser (Alp+Dox), Dox in LP‐Exos (LP‐Exos^Dox^), and finally Alp and Dox in LP‐Exos irradiated by laser (LP‐Exos^Alp+Dox^). CCK‐8 cytotoxicity analysis showed that compared with the PBS group, all of the others treatments damaged MGC803 viability with different extents. Compared with the treatments with single agent of either Dox or Alp, combined use of these two agents resulted in greater inhibition on cell viability. Meanwhile, utilization of LP‐Exos as carrier also promoted the damage effect to the MGC803 cells. Owing to the cooperation of the above two aspects, the LP‐Exos^Alp+Dox^ treatment thus showed the most effective viability inhibition in a dose‐dependent manner (**Figure** **5**a). Correspondingly, live/dead analysis highlighted the strong cell killing effects of the LP‐Exos^Alp+Dox^ treatment on cultured MGC803 cells, with most cells showing the red fluorescence (Figure 5b).

In addition to the 2D cell culture experiments, we extended the evaluations to 3D level, which could closely mimic native architecture of tumor tissues. To investigate the penetration and diffusion characteristics of LP‐Exos^Alp+Dox^, we prepared MGC803 cell spheroids to mimic tumor tissue and explored 3D interactions of cell spheroids with LP‐Exos^Alp+Dox^. CLSM imaging and corresponding line fluorescence intensity analysis of the LP‐Exos^Alp+Dox^ into MGC803 cell spheroids over a 24 h incubation showed time‐dependent increase in the penetration depth into spheroids (Figure 5c). The antitumor effects of the aforementioned six treatment types for the MGC803 cell spheroids were also detected. As shown in Figure 5d, terminal images for cell spheroids subjected to the six treatment types exhibited that the LP‐Exos^Alp+Dox^ treatment again outperformed other counterparts on suppressing the size of cell spheroids, with quantitative volume significantly decreased from 1.7 × 10^8^ (PBS group) to 2 × 10^7^ µm^3^.

To assess the antitumor effects of LP‐Exos^Alp+Dox^ in vivo, we generated MGC803‐derived tumor xenografts and subjected the mice to the six treatment types (tail vein for injection; 660 nm and 1 W laser for 3 min irradiation) (Figure 5e). Meanwhile, we carried out the treatment of dual‐loaded normal exosomes (N‐Exos^Alp+Dox^) for comparison of the therapeutic performance with LP‐Exos^Alp+Dox^, which could directly reveal the advantages induced by low pH treatment. Tumor volumes over time were measured every 2 days, and the data showed that all of these treatments exhibited different extents of tumor suppressive effects (Figure 5f). Owing to the targeting specificity, selective release, and combination therapy, LP‐Exos^Alp+Dox^ treatment exhibited the most significant inhibition on the tumor growth, compared to the others groups (Figure 5f and Figure S9 and Table S1, Supporting Information), and the tumor weight significantly decreased from 3.57 g (PBS group) to 0.48 g (Figure S10a, Supporting Information). Correspondingly, mice receiving LP‐Exos^Alp+Dox^ treatment all survived after 60 days, while mice in other groups had gradually died (Figure S10b, Supporting Information). To explore the microscopic properties underlying the observed tumor growth inhibition, we further measured markers of cell proliferation (Ki 67) and apoptosis (Cleaved Caspase‐3 and terminal deoxynucleotidyl transferase dUTP nick end labeling (TUNEL)) via immunohistochemical staining. Compared to PBS group, there was a decrease in the proliferation rate and an increase in the apoptosis rate for tumor cells treated with LP‐Exos^Alp+Dox^, which were less pronounced for the four other treatment types (Figure 5g). Moreover, note that above enhanced therapeutic outcomes of LP‐Exos^Alp+Dox^ were achieved with few abnormalities (Figure S10c,d, Supporting Information), demonstrating the biosafety of LP‐Exos^Alp+Dox^ in vivo, and the loading of Dox could exclude the possibility of tumor exosome's oncogenic risk.

To determine whether our dual‐loading strategy can be applied for other hydrophobic chemodrugs, we altered the chemodrug Dox to another chemodrug oridonin (Ori),^[^ ^38^ ^]^ and also examined LP‐Exos^Alp+Ori^ antitumor effects. In vitro, CCK‐8 and live/dead analyses showed that LP‐Exos^Alp+Ori^ treatment exhibited the strongest viability inhibition effects, compared to the others groups (Figure S11a,b, Supporting Information). In vivo, MGC803‐derived tumor xenografts were apparently suppressed by LP‐Exos^Alp+Ori^ treatment, not only reduced the tumor cells but also prolonged mice lifespan (Figures S11c–f and S12, Supporting Information), which again revealed the enhanced antitumor effects. The biosafety of LP‐Exos^Alp+Ori^ in vivo was also verified (Figure S13, Supporting Information). All demonstrated our LP‐Exos‐based dual‐loading strategy could be extended to other drug combinations for effective antitumor therapy.

### 2.6 Personalized LP‐Exos^Alp+Dox^ Exhibit Enhanced Tumor Cell Killing Effects in PDX Model Mice

**Figure 6:**
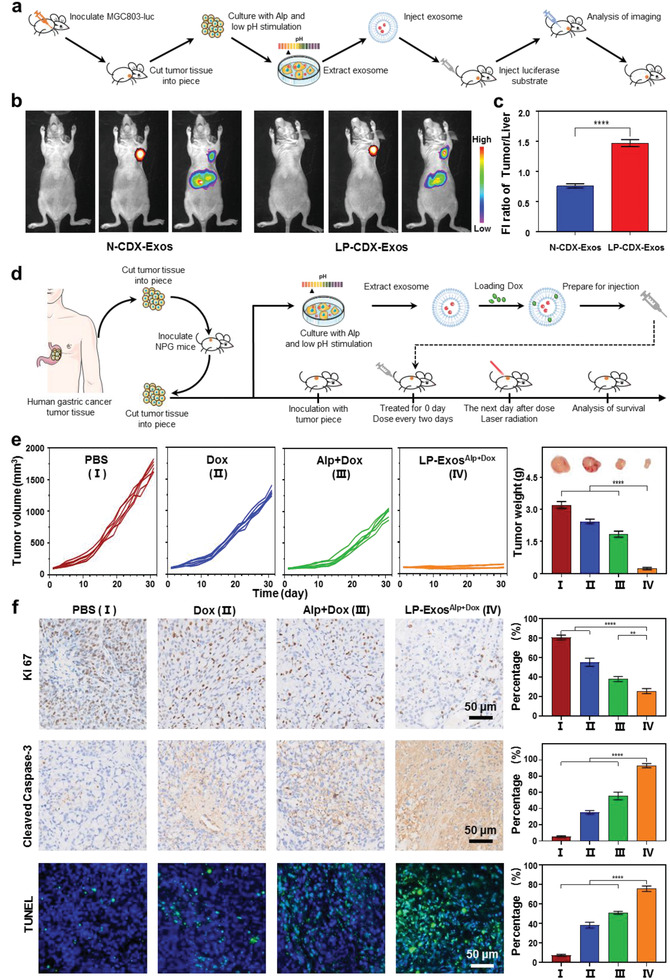
Personalized anticancer treatments based on LP‐Exos in PDX tumor models. a) Schematic illustration for experiments with CDX model mice. Low‐pH reprogramming applied to cells extracted from subcutaneous xenograft of MGC803‐luciferase nude mice. LP‐Exos released from reprogrammed cells were injected into the CDX mouse via the tail vein to examine tumor‐targeting performance. b) Bioluminescence and fluorescence imaging analyses of MGC803‐luciferase cells and exosomes, respectively, after injection of N‐CDX‐Exos (left panel) and LP‐CDX‐Exos (right panel) into the tail veins of mice bearing CDX tumors. c) Quantitative analysis of the tumor/liver fluorescence intensity (FI) ratio for CDX mice treated with N‐CDX‐Exos and LP‐CDX‐Exos. d) Schematic illustration of the PDX model generation and preparation of low‐pH reprogrammed, dual‐cargos loaded exosomes as anticancer agents: cells obtained from resected human gastric cancer tumor tissues were subcutaneously injected into the backs of mice, and then extracted cells were cultured either with or without low‐pH reprogramming, exosomes released from these cells were loaded with Alp and Dox, and injected into the PDX mouse via the tail vein to examine antitumor effects after laser irradiation. e) PDX tumor volumes after treatment with four diverse treatment types (PBS, Dox, Alp+Dox, LP‐Exos^Alp+Dox^) (left panel), with terminal weights and tumor images (right panel), each group contained six mice. f) Immunohistochemical analysis of tumor tissues and quantification of cell proliferation by Ki 67 (upper panel) and cell apoptosis by Cleaved Caspase‐3 (middle panel) and TUNEL (lower panel) in MGC803 tumor tissue at the end of treatment. Data in (c), (f) (*n* = 3), and (e) (*n* = 6) represent mean values ± SD. Statistical differences in (c) were determined by two‐tailed student's *t*‐test. Statistical differences in (e) and (f) were determined by one‐way ANOVA test. **p < 0.01, ^****^ *p* < 0.0001.

Note that all of the experiments of the present study have thus far used exosomes prepared from cultured cell lines. To promote medicine applications of exosome reprogramming for potential clinical therapy in the future, we next investigated the low‐pH‐programmed exosomes originated from patient tumor tissue for anti‐cancer treatment, i.e., personalized therapy. Before working directly with patient tissue, we worked with cell‐derived xenograft (CDX) model mice which were generated by injecting MGC803‐luciferase (MGC803‐luc) cells into the oxter of BALB/c null nude mice. After the CDX tumors developed, we excised part of the cancerous tissue and incubated the resected cells either with or without low‐pH programming. Exosomes released from these cell cultures were fluorescence‐labeled and then injected back into the CDX mouse via the tail vein. Luciferase substrate was also intraperitoneally injected to detect MGC803 cells, followed by bioluminescence and fluorescence imaging analyses (**Figure** **6**a). We found that MGC803 cells were concentrated in the oxter area, without transfer to other organs, and the low‐pH‐programmed exosomes (LP‐CDX‐Exos) from tumor cells were more highly concentrated in the CDX tumor area than the unprogrammed normal exosomes (N‐CDX‐Exos) (Figure 6b). Quantitative fluorescence intensity ratio also supported that the targeting efficiency of exosomes can be significantly improved through inclusion of the low‐pH reprogramming step during post‐resection cell culture (Figure 6c).

We then worked with PDX model mice, which we generated by subcutaneously inoculating the gastric tumor tissue obtained from a patient. We extracted the cells from the developed tumors, and cultured them with low‐pH reprogramming. After the dual‐loading, LP‐Exos^Alp+Dox^ were injected back into the PDX mouse via the tail vein to examine antitumor effects after laser irradiation (Figure 6d). We also treated the PDX mice with PBS, Dox alone, and Alp+Dox. As shown in Figure 6e, treatment with Dox alone failed to prominently inhibited tumor development, and this could be ameliorated with the combination of Alp (Alp+Dox). Once they were together loaded in LP‐Exos, we observed the most pronounced tumor inhibition effects in LP‐Exos^Alp+Dox^ group, with significantly decreased tumor weight from 3.21 g (PBS group) to 0.23 g. Correspondingly, mice receiving LP‐Exos^Alp+Dox^ treatment all survived after 70 days, while mice in other groups had gradually died (Figure S14a, Supporting Information). Staining for proliferation (Ki 67) and apoptosis (Cleaved Caspase‐3 and TUNEL) markers further reinforced the antiproliferative and apoptotic influence of the antitumor treatments, with the largest effects again observed for the LP‐Exos^Alp+Dox^ treatment (Figure 6f). Hematological analysis indicated the formulation of LP‐Exos shielded the cardiotoxicity of Dox, behaving outstanding biosafety (Figure S14b, Supporting Information), and hematoxylin and eosin staining showed that LP‐Exos^Alp+Dox^ had no remarkable side effects on normal tissues (Figure S14c, Supporting Information). Thus, exosomes sourced from resected tissues could be low‐pH reprogrammed, loaded with multiple therapeutic agents, and used to boost antitumor efficacy.

## 3 Discussion and Conclusion

In summary, we developed a way to reprogram tumor exosomes by low‐pH shock, which enabled improved tumor uptake and targeting efficiency. The underlying mechanism determined by lipidomics analysis and molecular dynamics simulations revealed a glycerolipid self‐aggregation‐based mechanism. We then demonstrated that these reprogrammed exosomes could be used as smart anticancer drug delivery vehicles to exert safe and enhanced antitumor effects, and offered an example of personalized therapy based on exosomes from cells of a gastric cancer patient in a matched PDX tumor model.

While the large majority of our efforts in the present study focused on altering exosome properties via low‐pH reprogramming, it bears mention that we also found hypoxia condition substantially improved the uptake efficiency of tumor exosomes by parent cancer cells, which implied feasible antitumor application prospects as the low‐pH treatment (Figure 2e and Figure S2b, Supporting Information). Considering that both acidity and hypoxia are the unique physical characteristics of tumor microenvironment, thus it is believable that manipulating the cell culture conditions to approximate some of the unique physical characteristics of tumor microenvironments can confer major performance benefits. Moreover, to extend the tumor microenvironment concept, it is worth noting that perhaps the selective expression or supplementation of exosomes with known or even patient‐specific ratios of cytokines, or other cell–cell interaction regulating biomolecules, can also educate exosomes for yet‐further increases in targeting efficiency and overall drug delivery performance. In addition, LP‐Exos can be further engineered elaborately to prolong their circulation time in vivo and strengthen the therapeutic performance and potential for clinical applications, such as employing CD47 “self” peptide or polyethylene glycol to modify its surface properties.^[^ ^39^, ^40^, ^41^ ^]^ In addition to exosomes, we also verified this acidic culture condition could affect tumor cell membranes, endowing them with enhanced targeting efficacy (Figure S15, Supporting Information). This phenomenon may also be attributed to the mechanism of GL's upregulation and aggregation, which can further extend the anticancer application using membrane‐based vesicles.

In this study, we have demonstrated extensions of these concepts to include multiple cell types (MGC803, HepG2) and a variety of chemically diverse antitumor agents (Dox, Ori, and Alp). Clinically, we hope this kind of anticancer exosomes can be suitable for postoperative combination therapy on a large variety of patient's primary carcinoma. Given the apparent ubiquity of exosome production by eukaryotic cells, besides the tumor‐related exosomes, one can envision education of exosomes from other cell types, e.g., stem cells or nerve cells, to improve delivery efficiency and achieve specific functionalization. As the loading of multiple agents into the LP‐Exos was extremely straightforward and had no effects on the gross morphological characteristics or performance attributes of the exosomes, we anticipate that further elaborations with a large variety of other agents and their potential combinations should be relatively easy to achieve for specific antitumor applications.

## Conflict of Interest

The authors declare no conflict of interest.

## Supporting information

Supporting Information

## Data Availability

The data that support the findings of this study are available from the corresponding author upon reasonable request.

## References

[1] C. Thery , L. Zitvogel , S. Amigorena , Nat. Rev. Immunol. 2002, 2, 569. 12154376 10.1038/nri855

[2] M. Tkach , C. Thery , Cell 2016, 164, 1226. 26967288 10.1016/j.cell.2016.01.043

[3] M. Simons , G. Raposo , Curr. Opin. Cell Biol. 2009, 21, 575. 19442504 10.1016/j.ceb.2009.03.007

[4] N. L. Syn , L. Wang , E. K. Chow , C. T. Lim , B. C. Goh , Trends Biotechnol. 2017, 35, 665. 28365132 10.1016/j.tibtech.2017.03.004

[5] A. A. Farooqi , N. N. Desai , M. Z. Qureshi , D. R. N. Librelotto , M. L. Gasparri , A. Bishayee , S. M. Nabavi , V. Curti , M. Daglia , Biotechnol. Adv. 2018, 36, 328. 29248680 10.1016/j.biotechadv.2017.12.010

[6] P. Vader , E. A. Mol , G. Pasterkamp , R. M. Schiffelers , Adv. Drug Delivery Rev. 2016, 106, 148. 10.1016/j.addr.2016.02.006 26928656

[7] L. Alvarez‐Erviti , Y. Seow , H. Yin , C. Betts , S. Lakhal , M. J. Wood , Nat. Biotechnol. 2011, 29, 341. 21423189 10.1038/nbt.1807

[8] L. A. Mulcahy , R. C. Pink , D. R. Carter , J. Extracell. Vesicles 2014, 3, 24641. 10.3402/jev.v3.24641 PMC4122821 25143819

[9] G. Morad , C. V. Carman , E. J. Hagedorn , J. R. Perlin , L. I. Zon , N. Mustafaoglu , T. E. Park , D. E. Ingber , C. C. Daisy , M. A. Moses , ACS Nano 2019, 13, 13853. 31479239 10.1021/acsnano.9b04397 PMC7169949

[10] H. Sun , J. Su , Q. Meng , Q. Yin , L. Chen , W. Gu , P. Zhang , Z. Zhang , H. Yu , S. Wang , Y. Li , Adv. Mater. 2016, 28, 9581. 27628433 10.1002/adma.201602173

[11] S. Tan , T. Wu , D. Zhang , Z. Zhang , Theranostics 2015, 5, 863. 26000058 10.7150/thno.11852 PMC4440443

[12] T. Yong , X. Zhang , N. Bie , H. Zhang , X. Zhang , F. Li , A. Hakeem , J. Hu , L. Gan , H. A. Santos , X. Yang , Nat. Commun. 2019, 10, 3838. 31444335 10.1038/s41467-019-11718-4 PMC6707218

[13] C. Xie , N. Ji , Z. Tang , J. Li , Q. Chen , Mol. Cancer 2019, 18, 83. 30954079 10.1186/s12943-019-0985-3 PMC6451295

[14] M. P. Bebelman , M. J. Smit , D. M. Pegtel , S. R. Baglio , Pharmacol. Ther. 2018, 188, 1. 29476772 10.1016/j.pharmthera.2018.02.013

[15] R. Kalluri , V. S. LeBleu , Science 2020, 367, eaau6977. 32029601 10.1126/science.aau6977 PMC7717626

[16] Q. Liang , N. Bie , T. Yong , K. Tang , X. Shi , Z. Wei , H. Jia , X. Zhang , H. Zhao , W. Huang , L. Gan , B. Huang , X. Yang , Nat. Biomed. Eng. 2019, 3, 729. 31110292 10.1038/s41551-019-0405-4

[17] H. Choudhry , A. L. Harris , Cell Metab. 2018, 27, 281. 29129785 10.1016/j.cmet.2017.10.005

[18] C. Shao , F. Yang , S. Miao , W. Liu , C. Wang , Y. Shu , H. Shen , Mol. Cancer 2018, 17, 120. 30098600 10.1186/s12943-018-0869-y PMC6087002

[19] H. W. King , M. Z. Michael , J. M. Gleadle , BMC Cancer 2012, 12, 421. 22998595 10.1186/1471-2407-12-421 PMC3488584

[20] M. Logozzi , D. Mizzoni , D. F. Angelini , R. Di Raimo , M. Falchi , L. Battistini , S. Fais , Cancers 2018, 10, 370. 10.3390/cancers10100370 PMC6210604 30301144

[21] Z. Boussadia , J. Lamberti , F. Mattei , E. Pizzi , R. Puglisi , C. Zanetti , L. Pasquini , F. Fratini , L. Fantozzi , F. Felicetti , K. Fecchi , C. Raggi , M. Sanchez , S. D'Atri , A. Care , M. Sargiacomo , I. Parolini , J. Exp. Clin. Cancer Res. 2018, 37, 245. 30290833 10.1186/s13046-018-0915-z PMC6173926

[22] M. Logozzi , E. Spugnini , D. Mizzoni , R. Di Raimo , S. Fais , Cancer Metastasis Rev. 2019, 38, 93. 30715644 10.1007/s10555-019-09783-8

[23] Y. Wang , D. S. Kohane , Nat. Rev. Mater. 2017, 2, 17020.

[24] W. Deng , W. Chen , S. Clement , A. Guller , Z. Zhao , A. Engel , E. M. Goldys , Nat. Commun. 2018, 9, 2713. 30006596 10.1038/s41467-018-05118-3 PMC6045614

[25] S. Huo , N. Gong , Y. Jiang , F. Chen , H. Guo , Y. Gan , Z. Wang , A. Herrmann , X. J. Liang , Sci. Adv. 2019, 5, eaaw6264. 31616782 10.1126/sciadv.aaw6264 PMC6774715

[26] D. Zhang , X. Qin , T. Wu , Q. Qiao , Q. Song , Z. Zhang , Biomaterials 2019, 197, 220. 30669014 10.1016/j.biomaterials.2019.01.024

[27] T. Wang , D. Wang , J. Liu , B. Feng , F. Zhou , H. Zhang , L. Zhou , Q. Yin , Z. Zhang , Z. Cao , H. Yu , Y. Li , Nano Lett. 2017, 17, 5429. 28753017 10.1021/acs.nanolett.7b02031

[28] A. Gao , B. Chen , J. Gao , F. Zhou , M. Saeed , B. Hou , Y. Li , H. Yu , Nano Lett. 2020, 20, 353. 31793787 10.1021/acs.nanolett.9b04012

[29] X. Pan , L. Bai , H. Wang , Q. Wu , H. Wang , S. Liu , B. Xu , X. Shi , H. Liu , Adv. Mater. 2018, 30, 1800180. 10.1002/adma.201800180 29672956

[30] C. Gong , J. Tian , Z. Wang , Y. Gao , X. Wu , X. Ding , L. Qiang , G. Li , Z. Han , Y. Yuan , S. Gao , J. Nanobiotechnol. 2019, 17, 93. 10.1186/s12951-019-0526-7 PMC6721253 31481080

[31] Y. Tian , S. Li , J. Song , T. Ji , M. Zhu , G. J. Anderson , J. Wei , G. Nie , Biomaterials 2014, 35, 2383. 24345736 10.1016/j.biomaterials.2013.11.083

[32] M. P. Bebelman , P. Bun , S. Huveneers , G. van Niel , D. M. Pegtel , F. J. Verweij , Nat. Protoc. 2020, 15, 102. 31836866 10.1038/s41596-019-0245-4

[33] S. Kourembanas , Annu. Rev. Physiol. 2015, 77, 13. 25293529 10.1146/annurev-physiol-021014-071641

[34] A. Janowska‐Wieczorek , M. Majka , J. Kijowski , M. Baj‐Krzyworzeka , R. Reca , A. R. Turner , J. Ratajczak , S. G. Emerson , M. A. Kowalska , M. Z. Ratajczak , Blood 2001, 98, 3143. 11698303 10.1182/blood.v98.10.3143

[35] S. J. Marrink , A. H. de Vries , A. E. Mark , J. Phys. Chem. B 2004, 108, 750.

[36] S. J. Marrink , H. J. Risselada , S. Yefimov , D. P. Tieleman , A. H. de Vries , J. Phys. Chem. B 2007, 111, 7812. 17569554 10.1021/jp071097f

[37] F. Rizzo , F. Polo , G. Bottaro , S. Fantacci , S. Antonello , L. Armelao , S. Quici , F. Maran , J. Am. Chem. Soc. 2017, 139, 2060. 28088858 10.1021/jacs.6b12247

[38] T. Zhen , C. F. Wu , P. Liu , H. Y. Wu , G. B. Zhou , Y. Lu , J. X. Liu , Y. Liang , K. K. Li , Y. Y. Wang , Y. Y. Xie , M. M. He , H. M. Cao , W. N. Zhang , L. M. Chen , K. Petrie , S. J. Chen , Z. Chen , Sci. Transl. Med. 2012, 4, 127ra38. 10.1126/scitranslmed.3003562 22461642

[39] P. L. Rodriguez , T. Harada , D. A. Christian , D. A. Pantano , R. K. Tsai , D. E. Discher , Science 2013, 339, 971. 23430657 10.1126/science.1229568 PMC3966479

[40] Y. Tang , X. Wang , J. Li , Y. Nie , G. Liao , Y. Yu , C. Li , ACS Nano 2019, 13, 13015. 31689086 10.1021/acsnano.9b05679

[41] J. S. Suk , Q. Xu , N. Kim , J. Hanes , L. M. Ensign , Adv. Drug Delivery Rev. 2016, 99, 28. 10.1016/j.addr.2015.09.012 PMC4798869 26456916

