# Supplementary material for: Tumor Exosomes Reprogrammed by Low pH Are Efficient Targeting Vehicles for Smart Drug Delivery and Personalized Therapy against their Homologous Tumor
Source: Adv Sci (Weinh). 2021 Mar 16;8(10):2002787. doi: 10.1002/advs.202002787 (PMC8132050; doi:10.1002/advs.202002787)
Supplement: Supplementary file 1 — Supporting Information [file ADVS-8-2002787-s001.pdf]

© 2021 Wiley-VCH GmbH

ADVANCED SCIENCE
Open Access

Supporting Information

for *Adv. Sci.*, DOI: 10.1002/advs.202002787

Tumor Exosomes Reprogrammed by Low pH are Efficient Targeting Vehicles for Smart Drug Delivery and Personalized Therapy Against Their Homologous Tumor

Changguo Gong, Xiao Zhang, Min Shi, Feng Li, Shuang Wang, Yan Wang, Yugang Wang, Wei Wei*, and Guanghui Ma**

Copyright WILEY-VCH Verlag GmbH & Co. KGaA, 69469 Weinheim, Germany, 2020.

Supporting Information

Tumor Exosomes Reprogrammed by Low pH are Efficient Targeting Vehicles for Smart Drug Delivery and Personalized Therapy Against Their Homologous Tumor

Changguo Gong, Xiao Zhang, Min Shi, Feng Li, Shuang Wang, Yan Wang, Yugang Wang, Wei Wei*, and Guanghui Ma**

Experimental Section

Materials and Methods

Mice

Balb/c nude mice (4-6 weeks, female) and NPG mice (4-6 weeks, female) were obtained from Vital River Laboratories (Beijing, China). The animal protocol was approved by the Institutional Animal Care and Use Committees at the Institute of Process Engineering, Chinese Academy of Sciences (approval ID: IPEAECA2018156). This study was performed in strict accordance with the Regulations for the Care and Use of Laboratory Animals and Guideline for Ethical Review of Animal (China, GB/T 35892-2018).

Cell culture

MGC803 cells (human gastric cancer cell line), HepG2 cells (human liver cancer cell line), and GES1 cells (human gastric mucosal epithelial cell line) were purchased from China Infrastructure of Cell Line Resource. NIH/3T3 cells (mouse fibroblast 3T3 cell line) were obtained from Cell Resource Center, IBMS, CAMS/PUMC. HUVEC cells (human umbilical vein endothelial cell line) were purchased from the American Type Culture Collection. MGC803-luciferase (MGC803-luc) cells were purchased from Liangkou Biotechnology Co., Ltd. (Shanghai, China). Cells were cultured in low-glucose Dulbecco's modified Eagle's medium (DMEM) with 10% fetal bovine serum (FBS) and 1% penicillin & streptomycin in a humidified CO_2 incubator with a 5% CO_2 atmosphere at 37 °C.

Cell stimulation

MGC803 cells were plated at a 10 cm petri dish for 24 h culture, and stimulated with various treatments for 30 min, including normal conditions without stimulation (N), ultraviolet irradiation stress treatment (UV; 40 W), low-pH culture medium treatment (LP; pH 4.0), high temperature treatment (HT; 40 °C), H_2O_2 treatment (H_2O_2; 250 μM), and hypoxia environment treatment (Hyp; 100% N_2). Then cells experienced an additional 24 h culture in the complete medium containing exosome-free serum, reaching 1.5×10^7 cells/dish. Exosome-free serum was prepared using centrifugation of FBS at 200,000 g at 4 °C for 4 h, and filtered the supernatant through a 0.22 μm filter.

Exosome extraction

Exosomes were prepared according to a typical protocol.[1] Briefly, 10 mL media supernatant was collected from a petri dish after cell stimulation, and underwent differential centrifugation: media was centrifuged at 300 g for 10 min, followed by 2,000 g for 10 min, 10,000 g for 30 min, and 100,000 g for 2 h at 4 °C.

Exosome characterization

TEM samples of exosomes were prepared according to a typical protocol,[1] and imaged by the HITACHI HT7700 transmission electron microscope (TEM). Size distributions for different exosomes were analyzed by nanoparticle tracking analysis (NTA; Zetaview, Particle Metrix) at 25 °C.

The expressions of CD9 and ALIX were evaluated by ProteinSimple Wes Capillary Western Blot analyzer. Briefly, total protein of exosomes was quantified using the bicinchoninic acid (BCA) assay kit.[2-4] Exosomes extracted from same amounts of cells (1.5×10^7) were diluted (1:2) with sample buffer (ProteinSimple) and the quantification

was performed using a 12-230 kDa 25-lane plate (PS-MK15, ProteinSimple) in WES according to the manufacturer's instructions. CD9 and ALIX were detected by their antibodies (ab92726 and ab117600, respectively) (Abcam Co., Cambridge, England).

Exosome's cell uptake

Various exosomes were labelled by DiD dye (Fanbo Biochemical Co., Beijing, China) through co-incubation at 37 °C for 1 h, and eluted with Exosome Spin Columns (Invitrogen Co., California, America). Cells were seeded in a confocal petri dish for 12 h, and added labelled exosomes at a concentration of 50 μg/mL. After 8 h incubation at 37 °C, cells were washed with PBS buffer, and cell membranes and nuclei were stained using Alexa Fluor™ 488-phalloidin (Thermo fisher Co., Massachusetts, America) (green), and DAPI (Fanbo Biochemical Co., Beijing, China) (blue), respectively. The cellular uptake of various exosomes was imaged using confocal laser scanning microscopy (CLSM, Leica TCS SP5). Average fluorescence intensity of DiD dye in cells filtered by a 300 mesh/meter nylon screen (Solarbio Biochemical Co., Beijing, China) was measured by flow cytometry (FACS, Beckman Coulter), using APC channel according to the manufacturer's instructions.

Exosome's tumor-targeting specificity *in vitro* and *in vivo*

MGC803 cells, GES1 cells, NIH/3T3 cells, and HUVEC cells were seeded in confocal petri dishes for 12 h. N-MGC803-Exos and LP-MGC803-Exos were labelled by DiD dye and added at a concentration of 50 μg/mL. After 8 h incubation, cellular uptake of N-MGC803-Exos or LP-MGC803-Exos by diverse cells were imaged using CLSM, and average fluorescence intensity of these cells was measured using flow

cytometry.

To observe the biodistribution of the exosomes *in vivo*, three types of exosomes were prepared: N-GES1-Exos, N-MGC803-Exos and LP-MGC803-Exos. Photosensitizer, Al (III) phthalocyanine chloride tetrasulfonic acid (Alp) (0.5 mg/mL) was used to label these exosomes by co-culture with the cells. Subcutaneous tumor xenografts were obtained by injecting MGC803 cells (1.5×10^7 cells) into oxter of Balb/c nude mice. After 16 days of feeding, 100 μL Alp-labelled exosomes were injected into MGC803 tumor-bearing female mice through the tail vein and their biodistribution was observed using an *in vivo* imaging system (Kodak FX Pro) at 2, 6, 12, 24, and 48 h. The terminal tumors and organs were excised and imaged. Frozen sections of tumor and organs at 48 h were prepared, and detected by automatic multispectral imaging system (PerkinElmer Vectra II) after DAPI staining.

LP-Exos dual-loading *in vitro*

Alp (0.5 mg/mL) was co-cultured with MGC803 cells under low-pH (4.0) condition. LP-Exos^{Alp} were extracted and incubated with doxorubicin (Dox) (Sangon Biotech Co., Ltd., Shanghai, China) (0.5 mg/mL) for 1 h at 37 °C. Dox contained fat-soluble anthracycline group, which allowed Dox's insertion into the lipid membrane by hydrophobic interaction, thus obtaining LP-Exos$^{Alp+Dox}$. The unloaded drugs were removed by elution with a 100 kDa ultrafiltration tube (Merck Millipore Co., Darmstadt, Germany). The loaded Dox concentration was calculated based on the absorbance intensity at 480 nm, and the loaded Alp concentration was at 674 nm by automatic microplate reader (Tecan Infinite M200). Drug-loading efficiency (DL) was calculated

according to equation 1:

$$DL(\%)=\frac{\text{Weight}_{\text{Drug}}}{\text{Weight}_{\text{Total protein}}}\times 100\% \qquad (1)$$

The dual-loaded LP-Exos$^{Alp+Dox}$ were also detected by CLSM and flow cytometry. The morphologies of LP-Exos$^{Alp+Dox}$ were imaged by TEM, the stabilities of size distribution and zeta potential were analyzed by NTA.

LP-Exos$^{Alp+Dox}$ drug release *in vitro*

A 660 nm wavelength laser at 1 W power was irradiated on LP-Exos$^{Alp+Dox}$ for 5 min to trigger the photosensitizer Alp excitation. Excited Alp transformed molecular oxygen into singlet oxygen. 9,10-Diphenylanthracene (DPA) (Sigma-aldrich Co., Missouri, America) was added into solution for detecting the absorbance intensity at 378 nm by automatic microplate reader, which were sensitive to the concentration of reactive oxygen species (ROS). The rupture of LP-Exos were imaged by TEM, and the Dox concentration in solution was monitored by automatic microplate reader.

In MGC803 cell experiments, MGC803 cells were co-cultured with LP-Exos$^{Alp+Dox}$ (50 μg/mL) for 4 h. ROS probe DCFH-DA (Sigma-aldrich Co., Missouri, America) was added into medium and incubated for 30 min. CLSM was used to monitor NIR-laser-triggered singlet oxygen production after 3 min laser irradiation (660 nm wavelength at 1 W power). The localizations of Alp and Dox before and after laser irradiation were also detected by CLSM.

Toxicity evaluation *in vitro*

To evaluate the toxicity of LP-Exos$^{Alp+Dox}$ on cells, CCK-8 (Beyotime Co., Shanghai, China) assay was used to determine the cytotoxicity. Briefly, MGC803 cells were

seeded in 96-well plates at a density of 1.0 × 10^4 cells in 100 μL of culture media for 12 h. Then Dox and Alp were added at a concentration ratio of 2:5, with the tested Dox concentrations being 0.2, 0.4, and 0.6 μg/mL and the concentrations of Alp being 0.5, 1.0, and 1.5 μg/mL. Six different treatment types at each concentration of Dox and Alp were tested, including PBS, Dox alone, Alp alone, Alp and Dox, LP-$Exos^{Dox}$, and LP-$Exos^{Alp+Dox}$. After 24 h incubation, CCK-8 solution was added and incubated for another 4 h. Percent viability was normalized according to the untreated cells.

The cytotoxicity was also measured by live/dead cell viability assay (Invitrogen Co., California, America). MGC803 cells were separately treated as described above. The cells were stained by live/dead staining working solution (Invitrogen Co., California, America) for 20 min at 37 °C and imaged by CLSM.

Penetration and growth inhibition of MGC803 cell spheroids *in vitro*

Tumor spheroids of MGC803 cells were prepared using a method as described previously.[5] To evaluate drug penetration in MGC803 cell spheroids, MGC803 cell spheroids were incubated with LP-$Exos^{Alp+Dox}$ (4.0 μg/mL Alp, 1.6 μg/mL Dox) for 24 h, and then analyzed by CLSM. To estimate the growth inhibition effect, the MGC803 cell spheroids were incubated with different treatments, including PBS, Dox alone (1.6 μg/mL), Alp alone (4.0 μg/mL), Alp and Dox (4.0 μg/mL Alp, 1.6 μg/mL Dox), LP-$Exos^{Dox}$ (1.6 μg/mL Dox), and LP-$Exos^{Alp+Dox}$ (4.0 μg/mL Alp, 1.6 μg/mL Dox) for 96 h. Growth inhibition of the tumor spheroids was monitored using an inverted phase microscope. The major (r_{max}) and minor (r_{min}) radii of each treated MGC803 cell

spheroids were determined, and the spheroid volume was calculated according to equation 2:

$$V = \frac{4}{3} \cdot \pi \times \left(\frac{r_{max}}{2} + \frac{r_{min}}{2}\right)^3 \quad (2)$$

Anticancer effect evaluations of LP-Exos$^{Alp+Dox}$ *in vivo*

For investigating *in vivo* antitumor effect of different treatments, MGC803-derived tumor xenografts were generated as described above, and tumor-bearing mice were treated after 16 days of feeding (i.e., day 0). Mice were injected with different treatments according to their body weight, including PBS, Dox alone (1.0 mg/kg), Alp alone (2.5 mg/kg), Alp and Dox (2.5 mg/kg Alp, 1.0 mg/kg Dox), LP-Exos^{Dox} (1.0 mg/kg Dox), N-Exos$^{Alp+Dox}$ (2.5 mg/kg Alp, 1.0 mg/kg Dox), and LP-Exos$^{Alp+Dox}$ (2.5 mg/kg Alp, 1.0 mg/kg Dox). Each group contained six mice. Mice were treated every 2 days *via* the tail vein, and the next day after treatment, the tumor area was irradiated by laser (660 nm, 1 W) for 3 min to excite Alp. Tumor volumes were calculated as equation 2, and tumor maximum allowable sizes were defined as 2,000 mm^3. Tumor growth inhibition (TGI) values were calculated for quantitative comparison, according to (1 - tumor volume in treatment group/tumor volume in PBS group) × 100%.

Immunohistochemical evaluations of LP-Exos$^{Alp+Dox}$ anticancer therapy

The above tumor tissues were collected and cut into thick sections after diverse treatments. Ki 67 detection was used for measuring the proliferation of tumor, and Cleaved Caspase-3 was used for measuring the apoptosis of tumor by automatic multispectral imaging system. A terminal deoxynucleotidyl transferase-mediated dUTP-biotin nick end labeling (TUNEL) apoptosis detection kit was also used,

according to the instruction provided by manufacturers (Merck Millipore Co., Darmstadt, Germany).

Biosafety evaluation of LP-Exos$^{Alp+Dox}$ anticancer therapy

To further evaluate the biosafety of LP-Exos$^{Alp+Dox}$ treatment *in vivo*, the serum levels of alanine aminotransferase (ALT), aspartate transaminase (AST), and alkaline phosphatase (ALP), lactate dehydrogenase (LDH), and urea nitrogen (BUN) were analyzed using an automated analyzer (Hitachi Ltd. Hitachi-917). The organs affected by LP-Exos$^{Alp+Dox}$ treatment were sliced and stained with hematoxylin and eosin.

CDX and PDX tumor model mice

Cell-derived xenograft (CDX) model mice were generated as follow: MGC803-luc cells (1.5×10^7 cells) were injected into the oxter of BALB/c null nude mice. After 22 days of feeding, part of the cancerous tissue was excised, cut into pieces and ground. A 70 μm cell filter was used to make a single-cell suspension. Then cells were cultured with Alp (0.5 mg/mL) either with or without low-pH treatment. A total of 100 μL of Alp-loaded N-CDX-Exos or LP-CDX-Exos were injected into the CDX mouse *via* the tail vein. Living image software (IVIS Living Image 4.2, PerkinElmer Ltd.) was used to acquire the data 5 min after intraperitoneal injection of D-luciferin (sodium salt) (15 mg/mL) into the mice. Bioluminescence imaging was conducted under autoexposure mode.

Patient-derived xenografts (PDX) model mice were generated as follow: human gastric cancer tumor tissues from a patient were resected and cut evenly into 5 mm × 5 mm pieces after removal of necrotic parts. After anesthesia of NPG mice, the tumor

piece was placed subcutaneously on the back. After 74 days of feeding, part of the cancerous tissue was excised, cut into pieces and ground. A 70 μm cell filter was used to make a single-cell suspension. Then cells were cultured as described above (dual-loading) with low-pH treatment. Four diverse treatments were tested and injected into the PDX mouse *via* the tail vein, including PBS, Dox alone (1.0 mg/kg), Alp and Dox (2.5 mg/kg Alp, 1.0 mg/kg Dox), and LP-Exos$^{Alp+Dox}$ (2.5 mg/kg Alp, 1.0 mg/kg Dox). Mice were treated every 2 days *via* the tail vein, and the next day after treatment, the tumor area was irradiated by laser (660 nm, 1 W) for 3 min to excite Alp. Tumor volumes were calculated as equation 2. Ethics Committee of Shanghai Tongren Hospital approved the study protocol, and the document number was AF/SC-07/02.1.

Lipidomics analysis

Lipidomics data were obtained from BiotechPack Technology Co. (Beijing, China).

Quartz crystal microbalance assay

Typical film dispersion method was used to synthesis N-Vesicles and LP-Vesicles.[6] DSPE-PEG2000 (Ruixi Biotechnology Co., Xian, China), sphingomyelin (SM) and triacylglycerol (TAG) (Sigma-aldrich Co., Missouri, America) were mixed (total 10 mg) in chloroform/methanol (1:1) solution according to the proportion of lipidomics data. The mixture was evaporated in a rotary evaporator at 37 °C to form a thin film. The thin film was dried under vacuum and rehydrated in the PBS solution (1 mL) under sonication at 37 °C. The mixture (10 mg/mL) was extruded through polycarbonate membrane (0.4 μm, 0.2 μm) (Avanti miniextruder) to form N-Vesicles and LP-Vesicles. Then 100 μL N-Vesicles were spin-coated on the Au chip to form a lipid membrane, N-

Vesicles or LP-Vesicles (60 ug/mL) were pumped to interact with the lipid membrane. After the curve reached a relatively stable level, the PBS solution was pumped and rinsed the unbound vesicles.

The cell membrane was prepared according to the method reported previously.[7-9] Briefly, the MGC803 and GES1 cells were first cultured at 37 °C for 24 h, then incubated with 0.1 mM azide-Cho for another 24 h. Next, cells were harvested and resuspended in HEPES buffer solution supplemented with 1% protease inhibitor cocktail. The cell suspension was destructed by IKAT18 basic ULTRA-TURRAX (IKA, Germany) and the consequent cellular membrane fragments were purified by discontinuous sucrose density gradient ultracentrifugation. Then 100 μL MGC803 or GES1 cell membrane fragments were spin-coated on the Au chip, LP-MGC803-Exos (60 ug/mL) were pumped to interact with the cell membrane. After the curve reached a relatively stable level, the complete medium was pumped and rinsed the unbound LP-MGC803-Exos.

Computer simulations

Computer simulations used the coarse-grained molecular dynamics technique, which extended the simulation scales of time and space to be appropriate to the study of vesicle-membrane systems. The models of two types of vesicles (N-Exos and LP-Exos) and a lipid membrane were constructed by charmm-gui platform,[10] with the most representative lipid subtypes of GPL, SL and GL. N-Exos and LP-Exos consisted of PE, SM, and TAG according to the proportion of lipidomics data, and the lipid membrane was identical with N-Exos. The diameter of vesicles was 20 nm, and the size of lipid

membrane was 50 × 50 nm^2. 1 μs equilibrium simulations were performed for all of the initial models. The terminal structures were applied in the subsequent production simulations. To test the exosome's targeting capacity, we modified the hydrophobicity of TAG's bead parameters to improve the interaction between vesicles and membrane for mimicking the specific receptors on the surface of MGC803 cells. These simulations were performed by GROMACS 5.1.2, in accordance with the standard procedures using the Dry Martini force field.[11] The characteristic distance and energy analyses were calculated by inner modules of software.

Statistical analysis

Statistical analyses were performed using GraphPad Prism 8.3.0 and SigmaPlot 10.0. Group sizes and definition of error bars were indicated in figure legends. Statistical analysis was performed using one-way ANOVA test. $P < 0.05$ was considered statistically significant, significance values were indicated as * $p<0.05$, ** $p<0.01$, *** $p<0.001$, and **** $p<0.0001$.

References

[1] C. Thery, S. Amigorena, G. Raposo, A. Clayton, *Curr. Protoc. Cell Biol.* **2006**, *Chapter 3*, Unit 3 22.

[2] C. Thery, K. W. Witwer, E. Aikawa, M. J. Alcaraz, J. D. Anderson, R. Andriantsitohaina, A. Antoniou, T. Arab, F. Archer, G. K. Atkin-Smith, D. C. Ayre, J. M. Bach, D. Bachurski, H. Baharvand, L. Balaj, S. Baldacchino, N. N. Bauer, A. A. Baxter, M. Bebawy, C. Beckham, A. Bedina Zavec, A. Benmoussa, A. C. Berardi, P. Bergese, E. Bielska, C. Blenkiron, S. Bobis-Wozowicz, E. Boilard, W. Boireau, A. Bongiovanni, F. E. Borras, S. Bosch, C. M. Boulanger, X. Breakefield, A. M. Breglio,

M. A. Brennan, D. R. Brigstock, A. Brisson, M. L. Broekman, J. F. Bromberg, P. Bryl-Gorecka, S. Buch, A. H. Buck, D. Burger, S. Busatto, D. Buschmann, B. Bussolati, E. I. Buzas, J. B. Byrd, G. Camussi, D. R. Carter, S. Caruso, L. W. Chamley, Y. T. Chang, C. Chen, S. Chen, L. Cheng, A. R. Chin, A. Clayton, S. P. Clerici, A. Cocks, E. Cocucci, R. J. Coffey, A. Cordeiro-da-Silva, Y. Couch, F. A. Coumans, B. Coyle, R. Crescitelli, M. F. Criado, C. D'Souza-Schorey, S. Das, A. Datta Chaudhuri, P. de Candia, E. F. De Santana, O. De Wever, H. A. Del Portillo, T. Demaret, S. Deville, A. Devitt, B. Dhondt, D. Di Vizio, L. C. Dieterich, V. Dolo, A. P. Dominguez Rubio, M. Dominici, M. R. Dourado, T. A. Driedonks, F. V. Duarte, H. M. Duncan, R. M. Eichenberger, K. Ekstrom, S. El Andaloussi, C. Elie-Caille, U. Erdbrugger, J. M. Falcon-Perez, F. Fatima, J. E. Fish, M. Flores-Bellver, A. Forsonits, A. Frelet-Barrand, F. Fricke, G. Fuhrmann, S. Gabrielsson, A. Gamez-Valero, C. Gardiner, K. Gartner, R. Gaudin, Y. S. Gho, B. Giebel, C. Gilbert, M. Gimona, I. Giusti, D. C. Goberdhan, A. Gorgens, S. M. Gorski, D. W. Greening, J. C. Gross, A. Gualerzi, G. N. Gupta, D. Gustafson, A. Handberg, R. A. Haraszti, P. Harrison, H. Hegyesi, A. Hendrix, A. F. Hill, F. H. Hochberg, K. F. Hoffmann, B. Holder, H. Holthofer, B. Hosseinkhani, G. Hu, Y. Huang, V. Huber, S. Hunt, A. G. Ibrahim, T. Ikezu, J. M. Inal, M. Isin, A. Ivanova, H. K. Jackson, S. Jacobsen, S. M. Jay, M. Jayachandran, G. Jenster, L. Jiang, S. M. Johnson, J. C. Jones, A. Jong, T. Jovanovic-Talisman, S. Jung, R. Kalluri, S. I. Kano, S. Kaur, Y. Kawamura, E. T. Keller, D. Khamari, E. Khomyakova, A. Khvorova, P. Kierulf, K. P. Kim, T. Kislinger, M. Klingeborn, D. J. Klinke, 2nd, M. Kornek, M. M. Kosanovic, A. F. Kovacs, E. M. Kramer-Albers, S. Krasemann, M. Krause, I. V. Kurochkin, G. D. Kusuma, S. Kuypers, S. Laitinen, S. M. Langevin, L. R. Languino, J. Lannigan, C. Lasser, L. C. Laurent, G. Lavieu, E. Lazaro-Ibanez, S. Le Lay, M. S. Lee, Y. X. F. Lee, D. S. Lemos, M. Lenassi, A. Leszczynska, I. T. Li, K. Liao, S. F. Libregts, E. Ligeti, R. Lim, S. K. Lim, A. Line, K. Linnemannstons, A. Llorente, C. A. Lombard, M. J. Lorenowicz, A. M. Lorincz, J. Lotvall, J. Lovett, M. C. Lowry, X. Loyer, Q. Lu, B. Lukomska, T. R. Lunavat, S. L. Maas, H. Malhi, A. Marcilla, J. Mariani, J. Mariscal, E. S. Martens-Uzunova, L. Martin-Jaular, M. C. Martinez, V. R. Martins, M. Mathieu, S. Mathivanan, M. Maugeri, L. K. McGinnis, M. J. McVey, D. G. Meckes, Jr., K. L.

Meehan, I. Mertens, V. R. Minciacchi, A. Moller, M. Moller Jorgensen, A. Morales-Kastresana, J. Morhayim, F. Mullier, M. Muraca, L. Musante, V. Mussack, D. C. Muth, K. H. Myburgh, T. Najrana, M. Nawaz, I. Nazarenko, P. Nejsum, C. Neri, T. Neri, R. Nieuwland, L. Nimrichter, J. P. Nolan, E. N. Nolte-'t Hoen, N. Noren Hooten, L. O'Driscoll, T. O'Grady, A. O'Loghlen, T. Ochiya, M. Olivier, A. Ortiz, L. A. Ortiz, X. Osteikoetxea, O. Ostergaard, M. Ostrowski, J. Park, D. M. Pegtel, H. Peinado, F. Perut, M. W. Pfaffl, D. G. Phinney, B. C. Pieters, R. C. Pink, D. S. Pisetsky, E. Pogge von Strandmann, I. Polakovicova, I. K. Poon, B. H. Powell, I. Prada, L. Pulliam, P. Quesenberry, A. Radeghieri, R. L. Raffai, S. Raimondo, J. Rak, M. I. Ramirez, G. Raposo, M. S. Rayyan, N. Regev-Rudzki, F. L. Ricklefs, P. D. Robbins, D. D. Roberts, S. C. Rodrigues, E. Rohde, S. Rome, K. M. Rouschop, A. Rughetti, A. E. Russell, P. Saa, S. Sahoo, E. Salas-Huenuleo, C. Sanchez, J. A. Saugstad, M. J. Saul, R. M. Schiffelers, R. Schneider, T. H. Schoyen, A. Scott, E. Shahaj, S. Sharma, O. Shatnyeva, F. Shekari, G. V. Shelke, A. K. Shetty, K. Shiba, P. R. Siljander, A. M. Silva, A. Skowronek, O. L. Snyder, 2nd, R. P. Soares, B. W. Sodar, C. Soekmadji, J. Sotillo, P. D. Stahl, W. Stoorvogel, S. L. Stott, E. F. Strasser, S. Swift, H. Tahara, M. Tewari, K. Timms, S. Tiwari, R. Tixeira, M. Tkach, W. S. Toh, R. Tomasini, A. C. Torrecilhas, J. P. Tosar, V. Toxavidis, L. Urbanelli, P. Vader, B. W. van Balkom, S. G. van der Grein, J. Van Deun, M. J. van Herwijnen, K. Van Keuren-Jensen, G. van Niel, M. E. van Royen, A. J. van Wijnen, M. H. Vasconcelos, I. J. Vechetti, Jr., T. D. Veit, L. J. Vella, E. Velot, F. J. Verweij, B. Vestad, J. L. Vinas, T. Visnovitz, K. V. Vukman, J. Wahlgren, D. C. Watson, M. H. Wauben, A. Weaver, J. P. Webber, V. Weber, A. M. Wehman, D. J. Weiss, J. A. Welsh, S. Wendt, A. M. Wheelock, Z. Wiener, L. Witte, J. Wolfram, A. Xagorari, P. Xander, J. Xu, X. Yan, M. Yanez-Mo, H. Yin, Y. Yuana, V. Zappulli, J. Zarubova, V. Zekas, J. Y. Zhang, Z. Zhao, L. Zheng, A. R. Zheutlin, A. M. Zickler, P. Zimmermann, A. M. Zivkovic, D. Zocco, E. K. Zuba-Surma, *J. Extracell. Vesicles* **2018**, *7*, 1535750.

[3] J. Kowal, G. Arras, M. Colombo, M. Jouve, J. P. Morath, B. Primdal-Bengtson, F. Dingli, D. Loew, M. Tkach, C. Thery, *Proc. Natl. Acad. Sci. U. S. A.* **2016**, *113*, E968.

[4] M. Mathieu, L. Martin-Jaular, G. Lavieu, C. Thery, *Nat. Cell Biol.* **2019**, *21*, 9.

[5] J. Friedrich, C. Seidel, R. Ebner, L. A. Kunz-Schughart, *Nat. Protoc.* **2009**, *4*, 309.

[6] M. Ismail, L. Ling, Y. Du, C. Yao, X. Li, *Biomaterials* **2018**, *163*, 76.

[7] F. Li, W. Nie, F. Zhang, G. Lu, C. Lv, Y. Lv, W. Bao, L. Zhang, S. Wang, X. Gao, W. Wei, H. Y. Xie, *ACS Cent. Sci.* **2019**, *5*, 796.

[8] G. Lu, C. Lv, W. Bao, F. Li, F. Zhang, L. Zhang, S. Wang, X. Gao, D. Zhao, W. Wei, H. Y. Xie, *Chem. Sci.* **2019**, *10*, 4847.

[9] F. Zhang, L. Zhao, S. Wang, J. Yang, G. Lu, N. Luo, X. Gao, G. Ma, H.-Y. Xie, W. Wei, *Adv. Funct. Mater.* **2018**, *28*, 1703326.

[10] Y. Qi, H. I. Ingolfsson, X. Cheng, J. Lee, S. J. Marrink, W. Im, *J. Chem. Theory Comput.* **2015**, *11*, 4486.

[11] C. Arnarez, J. J. Uusitalo, M. F. Masman, H. I. Ingolfsson, D. H. de Jong, M. N. Melo, X. Periole, A. H. de Vries, S. J. Marrink, *J. Chem. Theory Comput.* **2015**, *11*, 260.

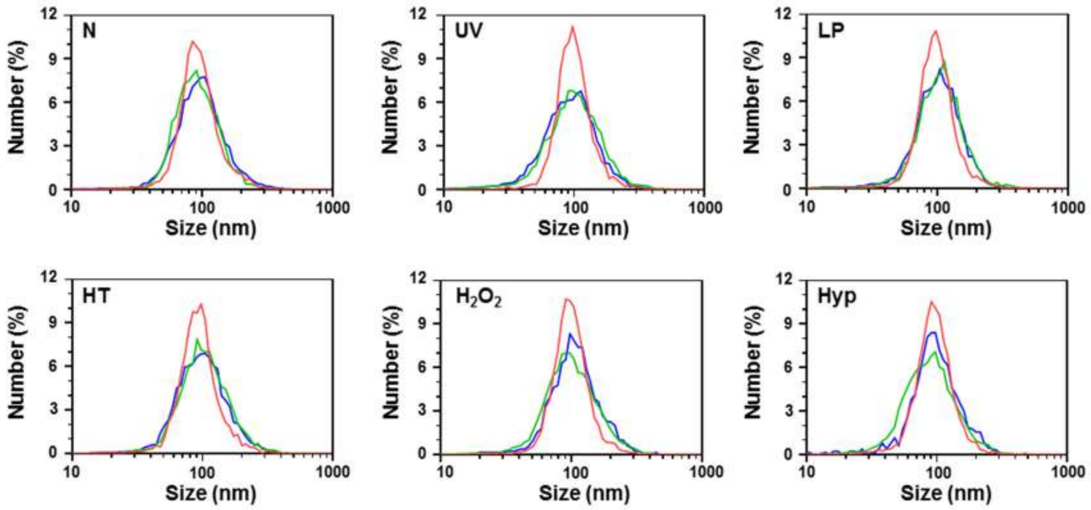

Figure S1. Size distributions of exosomes separated from the differentially treated MGC803 cells. Size distributions showing exosome separated from differently treated cells had similar sizes, which indicated these treatments did not alter exosomes' size (n = 3).

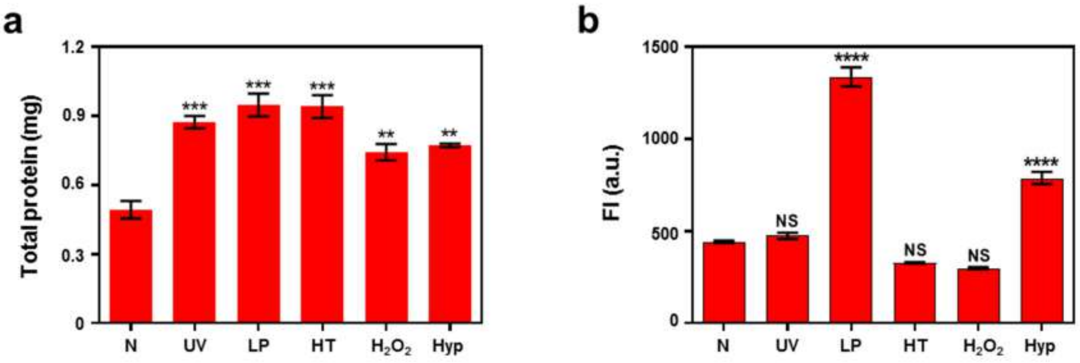

Figure S2. Evaluations of differentially treated HepG2 exosome types.

(**a**) BCA protein concentration detection for differentially treated HepG2 exosome types. Compared with the normal exosomes, there was significantly increased total exosome protein content in the samples of the various treatments.

(**b**) Fluorescence intensity (FI) of differentially treated HepG2 exosome types uptake by HepG2 cells, indicating that LP and Hyp treatments significantly improved the uptake efficiency.

Data in a and b represent mean values ± SD, n = 3. Statistical differences were determined by one-way ANOVA test. NS means no significant difference. ** $p<0.01$, *** $p<0.001$, **** $p<0.0001$.

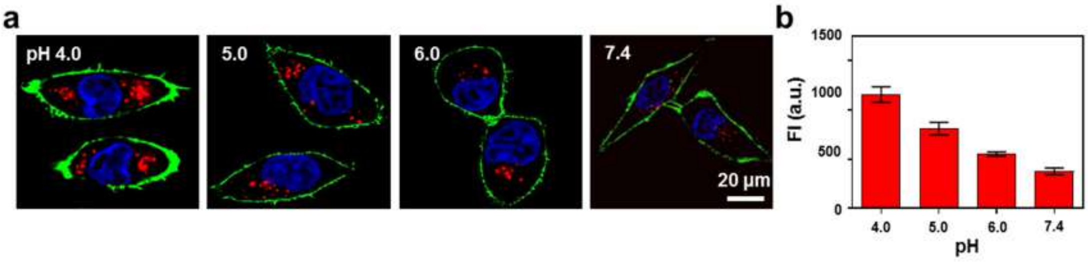

Figure S3. Effects of pH values on exosomes' uptake efficiency in LP treatment. (**a**) CLSM images of exosomes released by the MGC803 cells treated with different pH values uptake by MGC803 cells. Blue: nuclei; Green: membranes; Red: exosomes. (**b**) Quantitative fluorescence intensity (FI) of MGC803 exosomes uptake by MGC803 cells showing the uptake efficiency was gradually decreased with the pH value of culture medium increasing from 4.0 to 7.4.
Data in b represent mean values ± SD, n = 3.

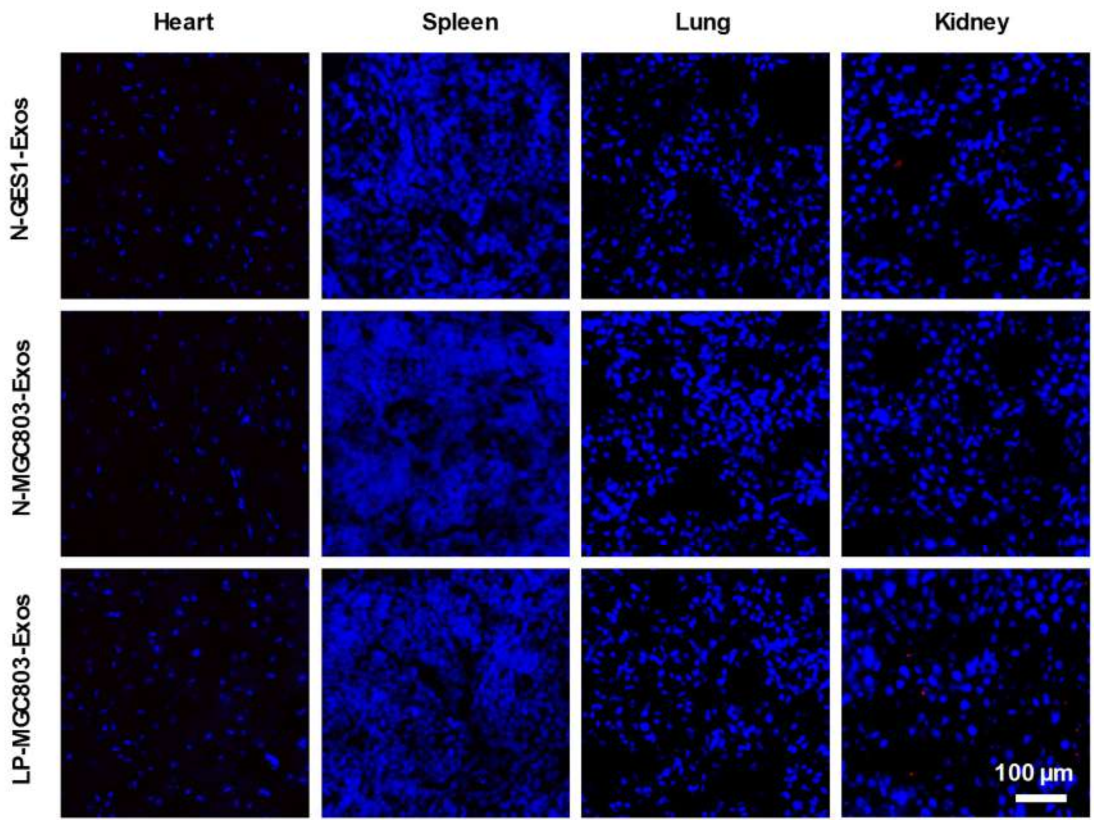

Figure S4. Targeting specificity of exosomes released by the low pH treated cells. Frozen sections of hearts, spleens, lungs, and kidneys after injecting N-GES1-Exos, N-MGC803-Exos, and LP-MGC803-Exos into MGC803 tumor bearing BALB/c null mice *in vivo*, showing that there were few exosomes existed in these organs, suggesting their tumor targeting specificity. Blue: nuclei; Red: exosomes.

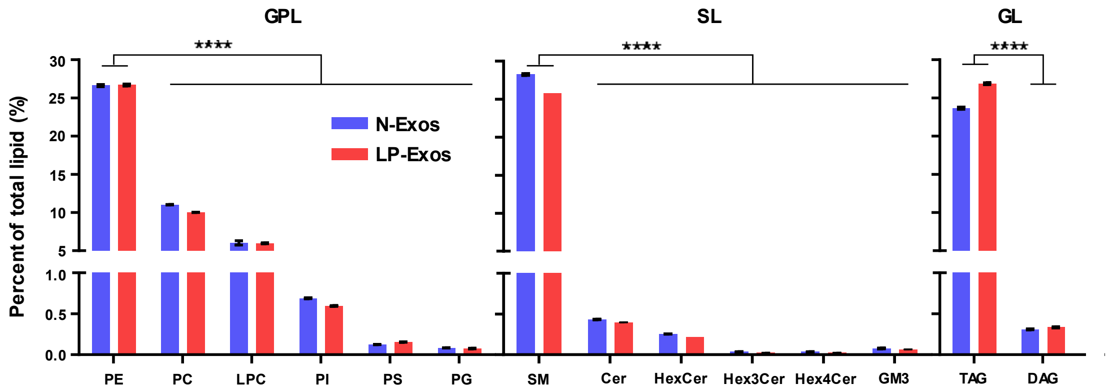

Figure S5. Lipidomics data for N-Exos and LP-Exos. More detailed comparison of lipid composition between N-Exos and LP-Exos. Blue: N-Exos; Red: LP-Exos. GPL mainly consisted of six lipid subtypes: phosphatidylethanolamine (PE), phosphatidylcholine (PC), lyso-PC (LPC), phosphatidylinositol (PI), phosphatidylserine (PS) and phosphatidylglycerol (PG), of which PE was the most dominant. SL was also mainly comprised six subtypes: sphingomyelin (SM), ceramide (Cer), hexosylceramide (HexCer), Hex3Cer, Hex4Cer and ganglioside GM3, of which SM was the most dominant. GL consisted of two subtypes: triacylglycerol (TAG) and diacylglycerol (DAG), of which TAG was the most dominant. These data provided specific lipid compositions for subsequent simulation calculations.

Data represent mean values ± SD, n = 3. Statistical differences were determined by two-way ANOVA test. **** $p<0.0001$.

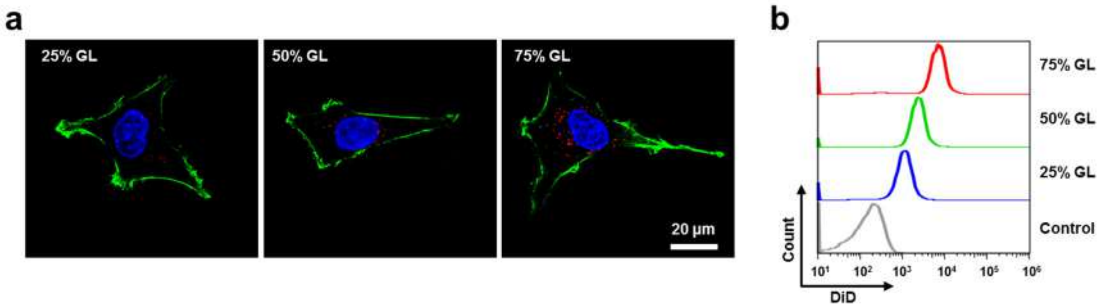

Figure S6. Vesicles with different ratios of GL uptake by MGC803 cells.
(**a**) CLSM images of vesicles with 25%, 50%, 75% GL uptake by MGC803 cells. Blue: cell nuclei; Green: cell membrane; Red: vesicles.
(**b**) Fluorescence intensity (from DiD labelled vesicles) in MGC803 cells detected by flow cytometry.
These data showed that the high GL ratio of vesicles enhanced the uptake efficiency by MGC803 cells.

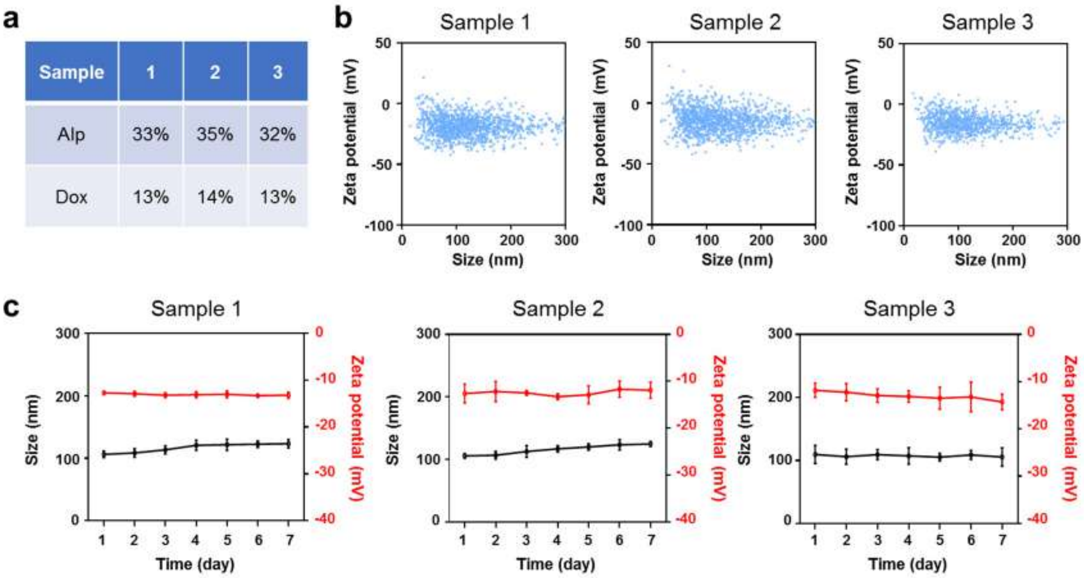

Figure S7. Reproducibility of the smart drug delivery platform LP-Exos$^{Alp+Dox}$.
(**a**) The drug loading ratios of Alp and Dox in exosomes.
(**b**) Size and zeta potential distributions of freshly prepared LP-Exos$^{Alp+Dox}$.
(**c**) The 7 days stability study.
Three isolated samples were prepared and measured according to the depicted engineering process, and these data demonstrated their superior reproducibility.
Data in c represent mean values ± SD, n = 3.

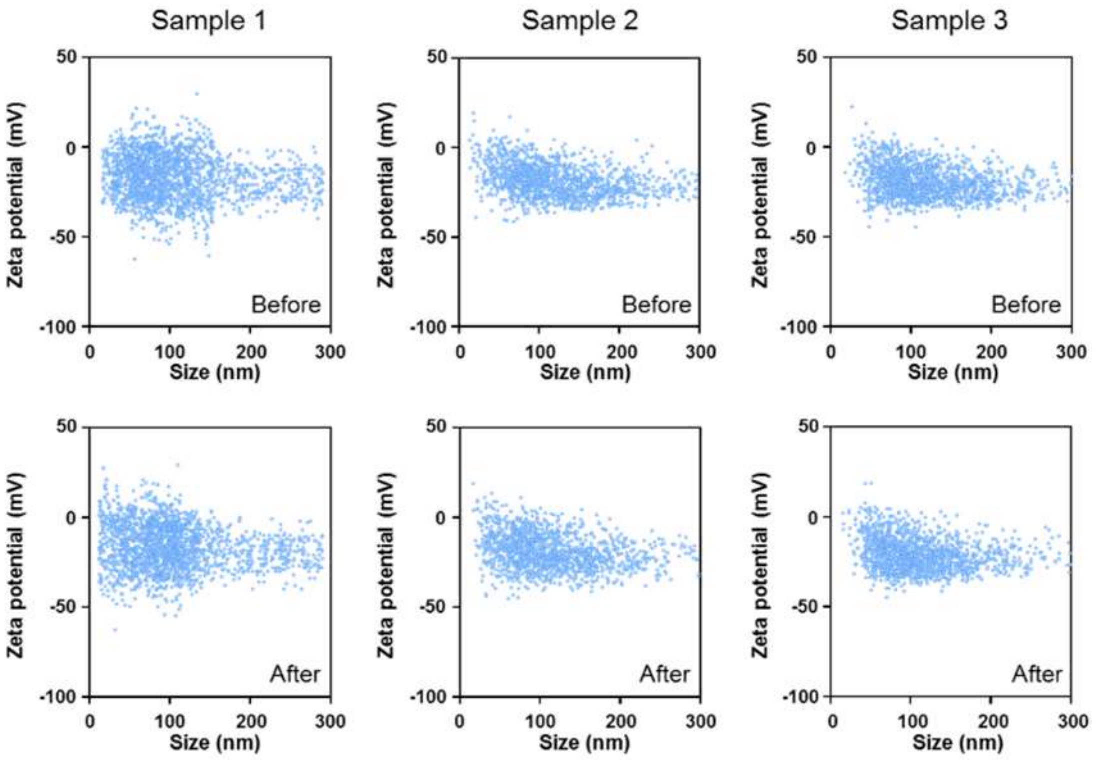

Figure S8. Lyophilized stability of the smart drug delivery platform LP-Exos$^{Alp+Dox}$. Size and zeta potential distributions measured by NTA of LP-Exos$^{Alp+Dox}$ before (upper panel) and after (bottom panel) lyophilization. Three isolated samples were measured to guarantee the reliability, and they shared similar sizes and zeta potentials, which demonstrated their lyophilized stability.

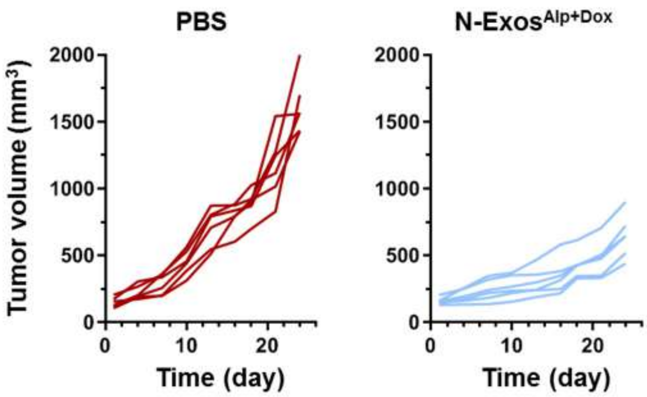

Figure S9. Evolutions of tumor volumes for MGC803-derived tumor xenograft treated by N-Exos$^{Alp+Dox}$. MGC803-derived tumor xenografts and the experimental procedures were following the method section of Figure 5e. Each group also contained six mice. Compared to the PBS group, the N-Exos$^{Alp+Dox}$ group exhibited moderate antitumor effect.

Group	PBS	Dox	Alp	Alp+Dox	LP-Exos^{Dox}	N-Exos$^{Alp+Dox}$	LP-Exos$^{Alp+Dox}$
TGI	0.00	0.24	0.25	0.45	0.46	0.61	0.87

Table S1. Tumor growth inhibition values of MGC803-derived tumor xenograft treated by different treatment types. Note that compared with LP-Exos$^{Alp+Dox}$, the TGI value of N-Exos$^{Alp+Dox}$ was compromised, which in turn demonstrated the greater benefit of LP-Exos compared to N-Exos.

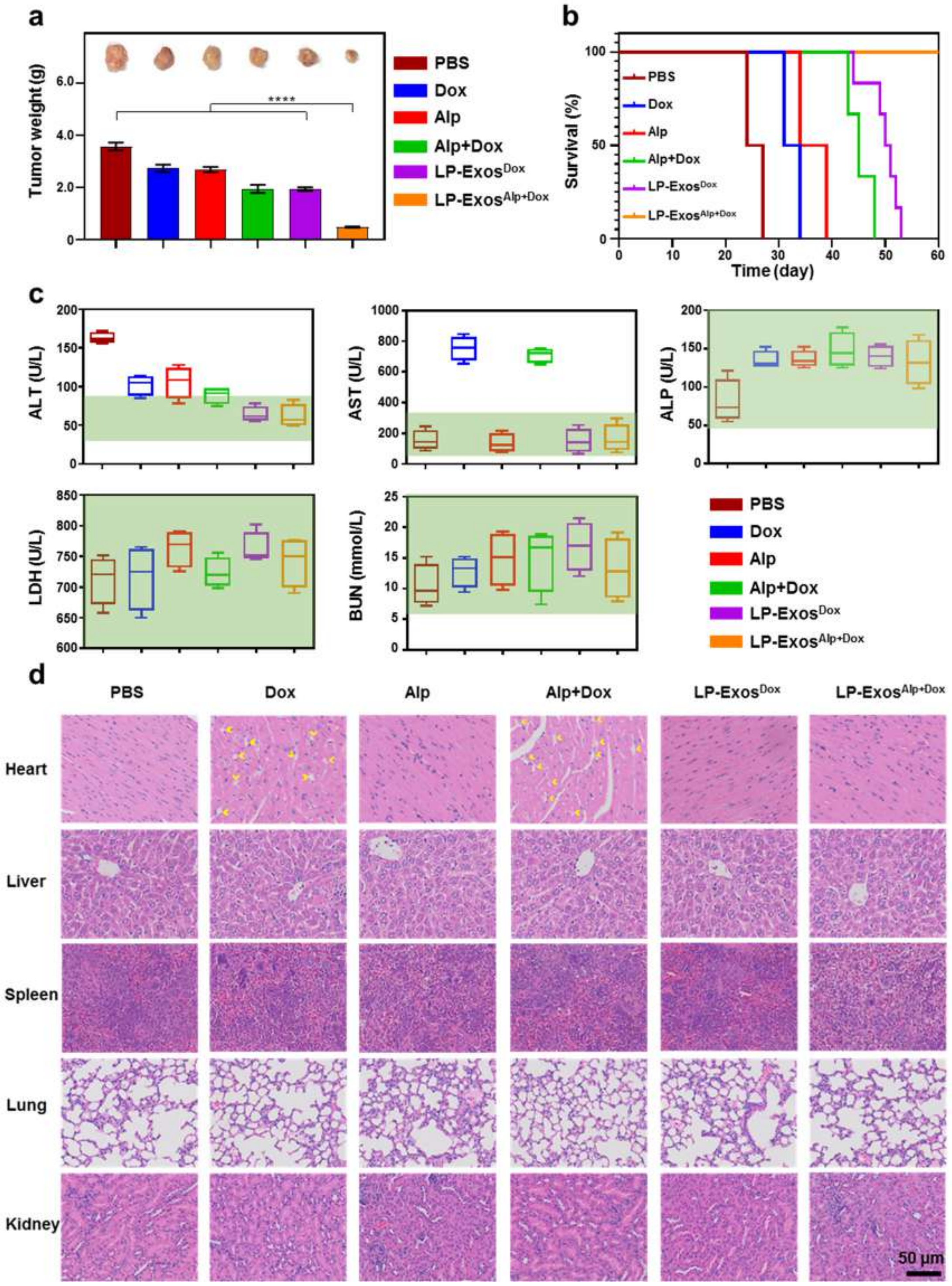

Figure S10. Anticancer therapy effects and biosafety of the LP-Exos$^{Alp+Dox}$ based smart drug release platform.

(**a**) Terminal tumor weights and images after treatment with six diverse treatment types (PBS, Dox, Alp, Alp+Dox, LP-Exos^{Dox}, and LP-Exos$^{Alp+Dox}$) for MGC803-derived tumor xenograft indicating LP-Exos$^{Alp+Dox}$ treatment significantly decreased tumor weight. Each group contained six mice.

(**b**) Mice survival curves with six diverse treatment types showing LP-Exos$^{Alp+Dox}$ treatment prolonged mice lifespan. Each group contained six mice.

(**c**) Hematological analysis of mice treated by six diverse treatments. Light green areas represent the normal range of different biosafety indicator. The results showed that the formulation of LP-Exos shielded the cardiotoxicity of Dox in the terms of biosafety indicator AST. Each group contained six mice.
(**d**) H&E-stained slice images of major organs (heart, liver, spleen, lung, and kidney) of six diverse treatment types (PBS, Dox, Alp, Alp+Dox, LP-Exos^{Dox}, and LP-Exos$^{Alp+Dox}$) for MGC803-derived tumor xenograft. The formulation of LP-Exos shielded the cardiotoxicity of Dox, and the LP-Exos$^{Alp+Dox}$ group had no metastatic foci, which demonstrated their safe use.
Data in a and c represent mean values ± SD, n = 6. Statistical differences were determined by one-way ANOVA test. **** $p<0.0001$.

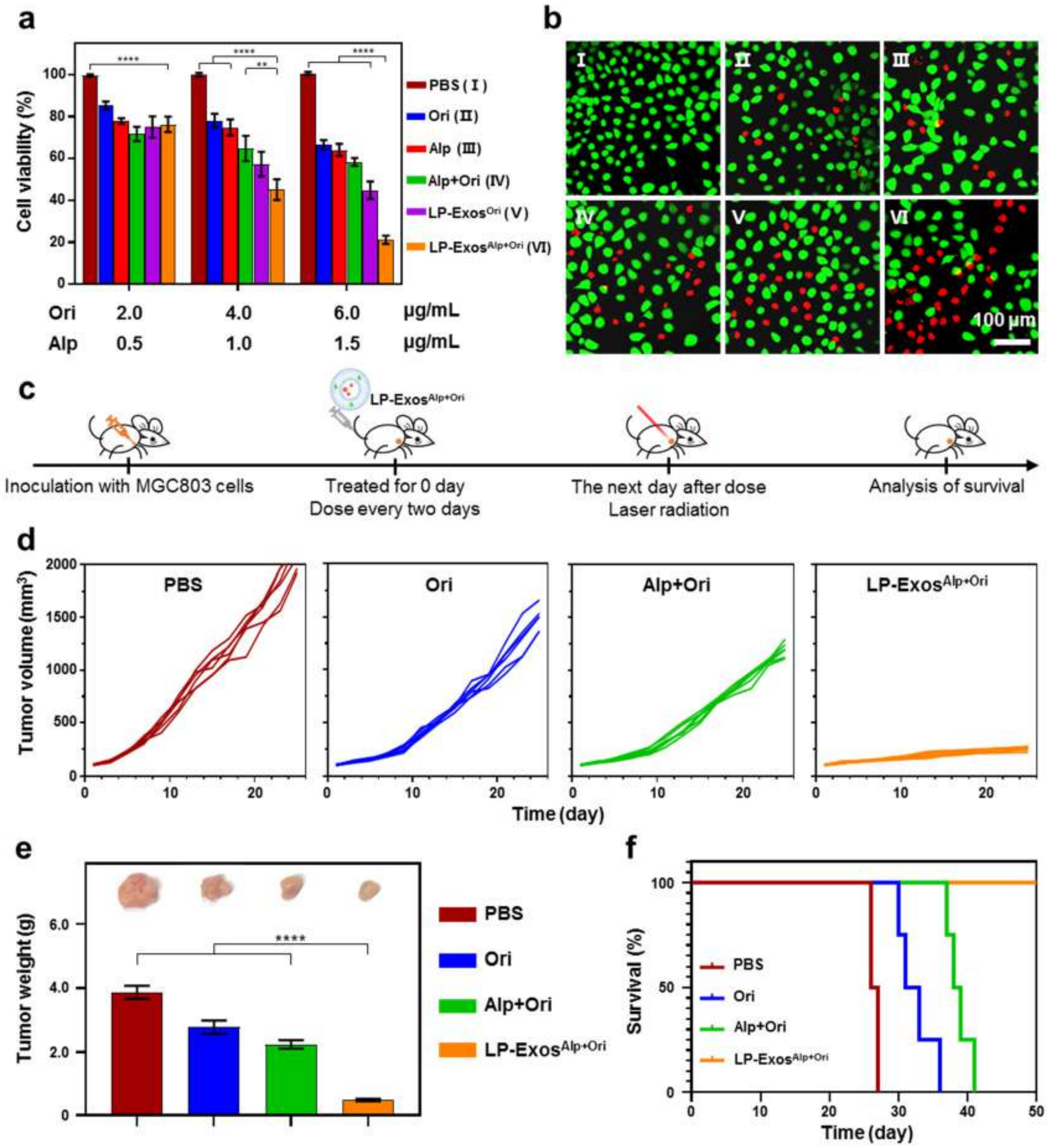

Figure S11. Evaluations of anticancer therapy effects of the LP-Exos$^{Alp+Ori}$ based smart drug release platform.

(**a**) CCK-8 cytotoxicity analysis of MGC803 cells given six treatment types (PBS, Ori, Alp, Alp+Ori, LP-Exos^{Ori}, and LP-Exos$^{Alp+Ori}$). Three different dose strengths were tested for each treatment. The doses of Ori and Alp were calculated from the loading rate in the approximate proportion of 4:1. The results showed LP-Exos$^{Alp+Ori}$ treatment had the most effective viability inhibition in a dose-dependent manner.

(**b**) Live/dead analysis after 24 h for cultured MGC803 cells given the six treatments, again highlighted the strong cell killing effects of the LP-Exos$^{Alp+Ori}$ treatment.

(**c**) Schematic diagram for constructing MGC803-derived tumor xenografts and the experimental design for four diverse treatment types (injected into mice *via* tail vein, and irradiated by 660 nm and 1 W laser).

(**d**) Evolution of tumor volumes for MGC803-derived tumor xenograft treated using four diverse treatments (PBS, Ori, Alp+Ori, and LP-Exos$^{Alp+Ori}$), each group contained six mice.

(**e**) Terminal tumor weights and images after treatment with four diverse treatment types for MGC803-derived tumor xenograft, indicating LP-Exos$^{Alp+Ori}$ treatment significantly decreased tumor weight. Each group contained six mice.
(**f**) Mice survival curves with four diverse treatment types showing LP-Exos$^{Alp+Ori}$ treatment prolonged mice lifespan. Each group contained six mice.
Data in a (n = 3) and e (n = 6) represent mean values ± SD. Statistical differences were determined by one-way ANOVA test. ** $p<0.01$, **** $p<0.0001$.

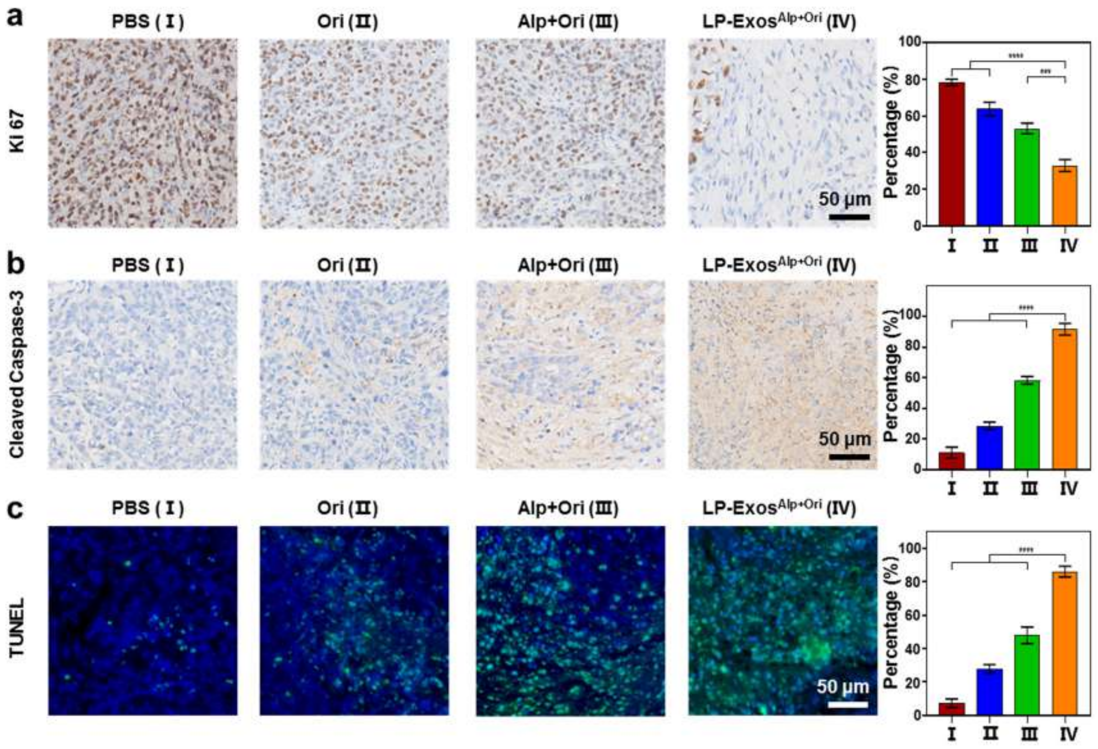

Figure S12. Immunohistochemical analysis of MGC803 tumor tissue after the LP-Exos$^{Alp+Ori}$ treatment.

(**a**) Comparisons on cell proliferation of MGC803 tumor tissues after four diverse treatments (PBS, Ori, Alp+Ori, and LP-Exos$^{Alp+Ori}$) by Ki 67.

(**b**) Comparisons on cell apoptosis of MGC803 tumor tissues after four diverse treatments by Cleaved Caspase-3.

(**c**) TUNEL analysis of MGC803 tumor tissues after four diverse treatments.

These results showed that there was a significant decrease in the proliferation rate and a significant increase in the apoptosis rate for tumor cells treated with LP-Exos$^{Alp+Ori}$, revealing its superior anti-tumor effects.

Data in a, b and c represent mean values ± SD, n = 3. Statistical differences were determined by one-way ANOVA test. *** $p<0.001$, **** $p<0.0001$.

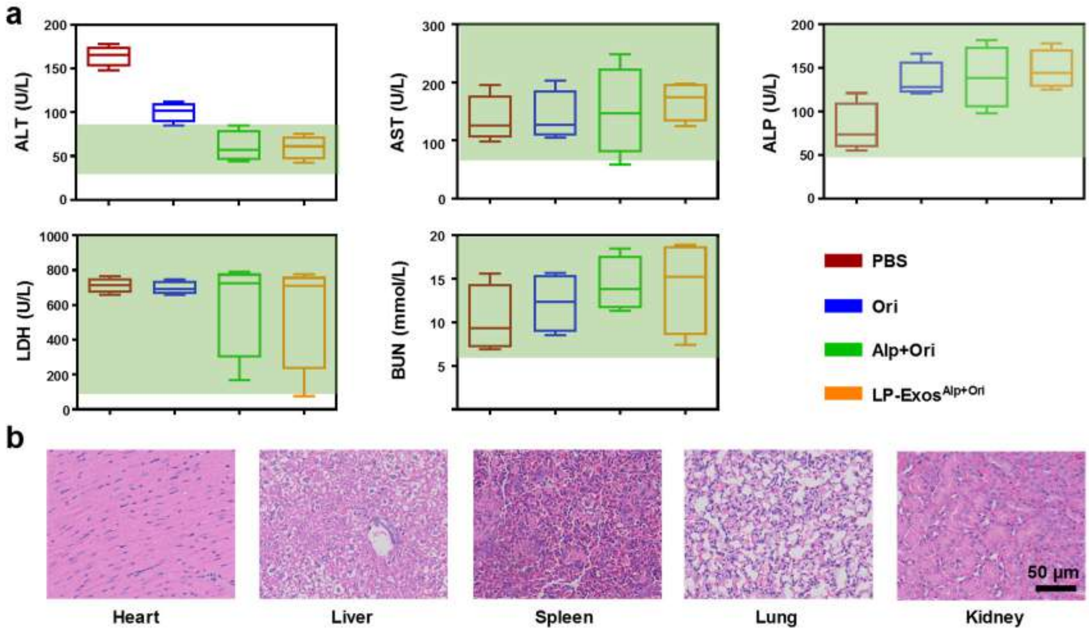

Figure S13. Evaluations of biosafety of the LP-Exos$^{Alp+Ori}$ treatment.

(**a**) Hematological analysis of mice treated by four diverse treatments. Light green areas represent the normal range of different biosafety indicator. The results indicated that LP-Exos$^{Alp+Ori}$ treatment had no side effects. Each group contained six mice.

(**b**) H&E-stained slice images of major organs (heart, liver, spleen, lung, and kidney) of the LP-Exos$^{Alp+Ori}$ treatment suggesting LP-Exos$^{Alp+Ori}$ treatment was benign for these organs.

Data in a represent mean values ± SD, n = 6.

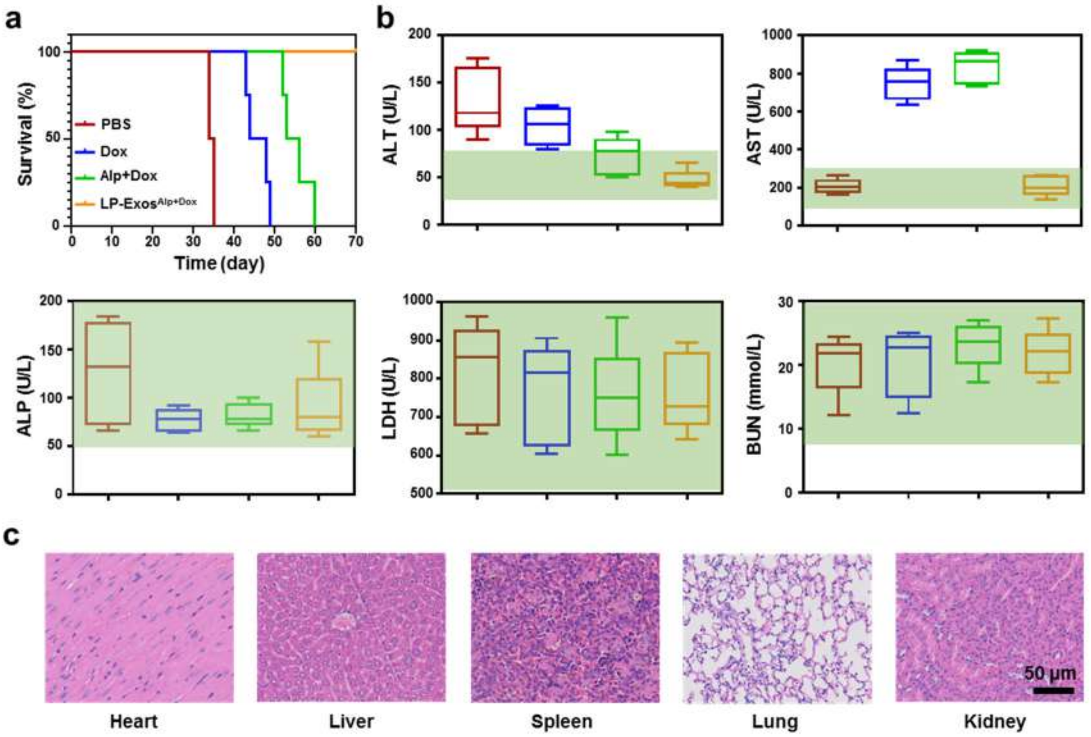

Figure S14. Evaluations of personalized anticancer effects and biosafety of the LP-Exos$^{Alp+Dox}$ in PDX tumor models.

(**a**) Mice survival curves with four diverse treatment types (PBS, Dox, Alp+Dox, and LP-Exos$^{Alp+Dox}$) showing LP-Exos$^{Alp+Dox}$ treatment prolonged mice lifespan. Each group contained six mice.

(**b**) Hematological analysis of mice treated by four diverse treatments. Light green areas represent the normal range of different biosafety indicator. The results again showed that the formulation of LP-Exos shielded the cardiotoxicity of Dox in the terms of biosafety indicator AST. Each group contained six mice.

(**c**) H&E-stained slice images of major organs (heart, liver, spleen, lung, and kidney) of LP-Exos$^{Alp+Doxi}$ treatment suggesting LP-Exos$^{Alp+Dox}$ treatment was benign for these organs.

Data in b represent mean values ± SD, n = 6.

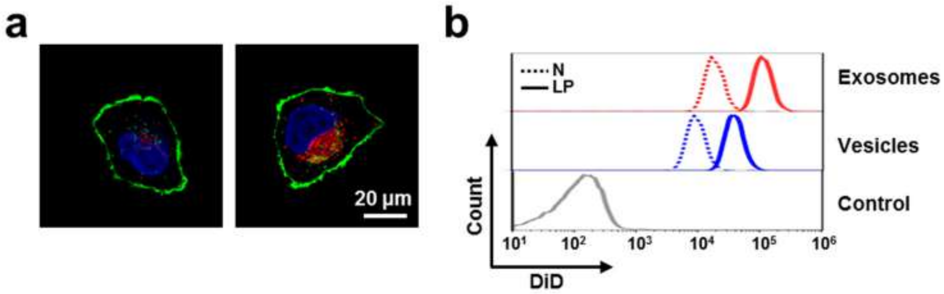

Figure S15. Targeting efficacy of MGC803 membrane-component vesicles for MGC803 cells.

(**a**) CLSM images of N-M-Vesicles (left panel) and LP-M-Vesicles (right panel) endocytosed by MGC803 cells. Blue: cell nuclei; Green: cell membrane; Red: exosomes.

(**b**) Comparison of fluorescence intensity of N-M-Vesicles *vs* LP-M-Vesicles, N-Exos *vs* LP-Exos in MGC803 cells by flow cytometry.

These data demonstrated that low pH treatment could also reprogram tumor cell membrane for enhanced targeting efficacy.
